# Spatiotemporal trends in temperature and rainfall within the coffea arabica landscapes of Gimbo and Decha districts in the Kafa Zone, Southwest Ethiopia

**DOI:** 10.1038/s41598-026-54593-y

**Published:** 2026-05-31

**Authors:** Amare Amsalu, Ademe Tizazu, Sintayehu Teka Bedhadha, Dessalegn Obsi Gemeda

**Affiliations:** 1 Department of Natural Resource Management, College of Agriculture and Natural Resource, Bonga University, Bonga, Ethiopia; 2 Department of Soil Resource and Watershed Management, College of Agriculture and Natural Resource, Bonga University, Bonga, Ethiopia; 3 Department of Geography and Environmental Studies, College of Social Sciences and Humanities, Jimma University, Jimma, Ethiopia; 4 Department of Natural Resource Management, College of Agriculture and Veterinary Medicine, Jimma University, Jimma, Ethiopia

**Keywords:** Climate variability, Coffea arabica, Precipitation concentration index, Regression kriging, Southwest ethiopia, Climate sciences, Ecology, Ecology, Environmental sciences

## Abstract

Climate change poses escalating risks to rain-fed agricultural systems, particularly for Coffea arabica, a perennial crop with narrow bioclimatic requirements that supports the livelihoods and ecological heritage of Ethiopia. This study evaluates spatiotemporal trends in temperature and rainfall across the Gimbo and Decha districts from 1990 to 2022, with the specific objectives of characterizing shifts in thermal regimes, assessing rainfall distribution and concentration, and mapping the spatial heterogeneity of climatic trends. Historical records from ten meteorological stations were integrated with CHIRPS and ERA5-Land datasets, and analyzed using the Mann-Kendall test, Sen’s slope estimator, Innovative Trend Analysis, Precipitation Concentration Index, and regression kriging for spatial interpolation. Results reveal a coherent warming signal with annual mean temperatures increasing by +0.02°C year⁻^1^ (p < 0.001). Maximum temperature rise significantly during the dry months (January-March), while minimum temperature increased notably during the main rainy season (June-July), suggesting a gradual compression of the diurnal temperature range. Annual rainfall exhibited a modest upward trend (+2.69 mm year⁻^1^, p = 0.016), yet seasonal precipitation is highly concentrated (PCI > 20 in 75.8% of Kiremt and 61.8% of Belg) and marked by noticeable interannual irregularity, including sharp oscillations between extreme dry and wet periods. Spatial interpolation highlighted topographically mediated microclimatic gradients, with warmer southern parts contrasting with cooler northern and eastern zones. Collectively, these patterns indicate a shifting environmental baseline characterized by escalating thermal stress and hydro-climatic unpredictability, which may progressively challenge coffee phenological synchronization and yield stability. The study forms a spatially explicit, ground-validated climatic baseline data that can inform targeted, landscape-level adaptation strategies, and serve as a foundational dataset for integrating process-based crop models and climate-resilient planning in this highly significant coffee-producing region.

**Supplementary Information:**

The online version contains supplementary material available at 10.1038/s41598-026-54593-y.

## Introduction

### Background of study

Climate change represents one of the most pressing challenges of the 21^st^ century, exerting profound and widespread impacts on global ecosystems, economic stability, and societal well-being. The agricultural sector, a cornerstone of food security and livelihoods for billions, is highly vulnerable to these changes. Rising global temperatures, altered precipitation regimes, and the increasing frequency of extreme weather events are projected to challenge crop productivity and disrupt established farming systems^1^. This vulnerability is not uniformly distributed, it is magnified in regions where economies are agrarian, infrastructure is limited, and adaptive capacity is constrained^2^. Consequently, understanding and mitigating the impacts of climate variability on agriculture has become a critical imperative for sustainable development.

Africa stands on the frontline of this climatic crisis, facing disproportionate risks due to its high socio-economic vulnerability and intrinsic dependence on climate-sensitive rain-fed agriculture^3^. Within the continent, Sub-Saharan Africa is experiencing some of the most pronounced climatic shifts, including accelerated warming and increased rainfall variability^4^. These changes manifest as erratic growing seasons, intensified drought and flood cycles, and the destabilization of agricultural systems historically calibrated to stable climatic norms. Such disruptions pose significant threats to rural livelihoods, food security, and economic resilience, underscoring the need for location-specific evidence to inform adaptive responses^5^.

Ethiopia is one of the most vulnerable country to climate change as its economy and the well-being of much of its population are closely tied to climate-sensitive sectors, predominantly subsistence agriculture and livestock production^6^. Climate projections indicate potential temperature increases of 0.9-1.1 °C by 2030, 1.7-2.1 °C by 2050, and 2.7-3.4 °C by 2080^7^, alongside shifts in rainfall distribution that could alter water availability and extreme event frequencies. These trajectories are expected to progressively reshape the country’s agricultural landscape. Among the crops at risk, Coffea arabica holds particular significance. As Ethiopia’s primary export commodity and a vital income source for millions of smallholder households^8^, coffee is deeply embedded in the nation’s cultural and economic fabric^9^. As a perennial species with narrow bioclimatic requirements, Ethiopian coffee exhibits high sensitivity to temperature, which can influence physiological development, yield potential, and bean quality^10^.

Emerging research highlights several pathways through which climate variability may influence Ethiopian coffee production. First, bioclimatic suitability models consistently project a contraction of optimal growing areas at lower elevations, with potential upward shifts constrained by soil quality, land tenure, and topographic limitations^11^. Second, physiological responses to warming remain an active area of investigation. Process-based modeling and field observations suggest that elevated temperatures and altered water availability may affect key developmental stages, including flowering synchrony and fruit set, potentially reducing yields under unmanaged conditions^12^. However, quantitative yield responses are highly context-dependent, mediated by agroecological factors such as shade cover, soil moisture retention, and microclimate buffering. Third, shifts in rainfall timing and intensity are assumed to disrupt phenological cycles, particularly the rainfall-triggered flowering synchronization critical for uniform harvests. Thus, while modeled projections and physiological studies indicate significant vulnerability, on-farm outcomes will depend on the interplay between climatic stressors and localized management practices.

A compounding factor is the potential expansion and intensification of coffee pests and diseases under warmer, more humid conditions^13^. Pathogens such as coffee leaf rust (Hemileia vastatrix) and pests like the coffee berry borer (Hypothenemus hampei) exhibit temperature- and moisture-dependent life cycles, and climatic shifts may alter their geographic ranges and outbreak frequencies^14^. While empirical evidence confirms increased biotic pressure in several tropical regions, the magnitude of yield loss in Ethiopian smallholder systems remains dependent on local agroecological conditions and integrated pest management adoption. The convergence of abiotic stressors and biotic pressures, however, is widely projected to amplify socio-economic risks, including income volatility for smallholders and potential destabilization of coffee-dependent value chains^15^.

The Kafa Zone represents a critical microcosm where these broader climatic threats intersect with localized socio-ecological dynamics. It is recognized as part of the Eastern Afromontane Biodiversity Hotspot, the region hosts the genetic diversity of wild Coffea arabica and supports smallholder coffee farming as a dominant livelihood^16^. Coffee is typically cultivated within diverse agroforestry systems that contribute to household resilience and forest conservation. However, this unique system is increasingly exposed to climate variability^17^. Local observations indicate increasing stress on agricultural production, raising concerns for both farmer livelihoods and the conservation of irreplaceable genetic resources^18^^,^^19^. However, translating these observations into targeted adaptation requires rigorous, spatially explicit climate trend data that can be directly linked to crop-relevant environmental thresholds.

Unlike previous national-scale assessments, this study provides high-resolution, station-integrated, and spatially explicit climate trend analysis at the district scale, enabling more precise linkage to coffee agroecological conditions. Furthermore, bridging observed climatic trends with process-based indicators, such as crop water deficit metrics and evaporative demand, is essential for moving from descriptive trend analysis to actionable agronomic insight^20^. Robust, long-term trend analysis at the local scale is therefore a fundamental prerequisite for assessing system-specific vulnerabilities, validating farmer perceptions, and designing evidence-based adaptation strategies.

This study addresses this gap by conducting a detailed spatiotemporal analysis of climate change and variability within the coffee-growing landscapes of the Gimbo and Decha districts in the Kafa Zone. Specifically, this research quantifies historical trends (1990-2022) in monthly, seasonal, and annual temperature and rainfall parameters. By applying rigorous statistical trend detection and spatial interpolation techniques, the current study aims to: (1) quantify the magnitude and statistical significance of trends in minimum, maximum, and mean temperatures; (2) analyze shifts in rainfall amount, temporal distribution, and concentration indices; and (3) map the spatial heterogeneity of these climatic trends across the study area. This study establishes an empirical climate baseline for integration with process-based crop models and agronomic assessments^20^. This foundation is vital for subsequent work evaluating coffee yield sensitivity, characterizing smallholder vulnerability, and co-designing resilient, context-specific adaptation pathways to safeguard the future of coffee farming in this important Ethiopian landscape.

## Methodology

### Description of the study area

**Fig. 1:**
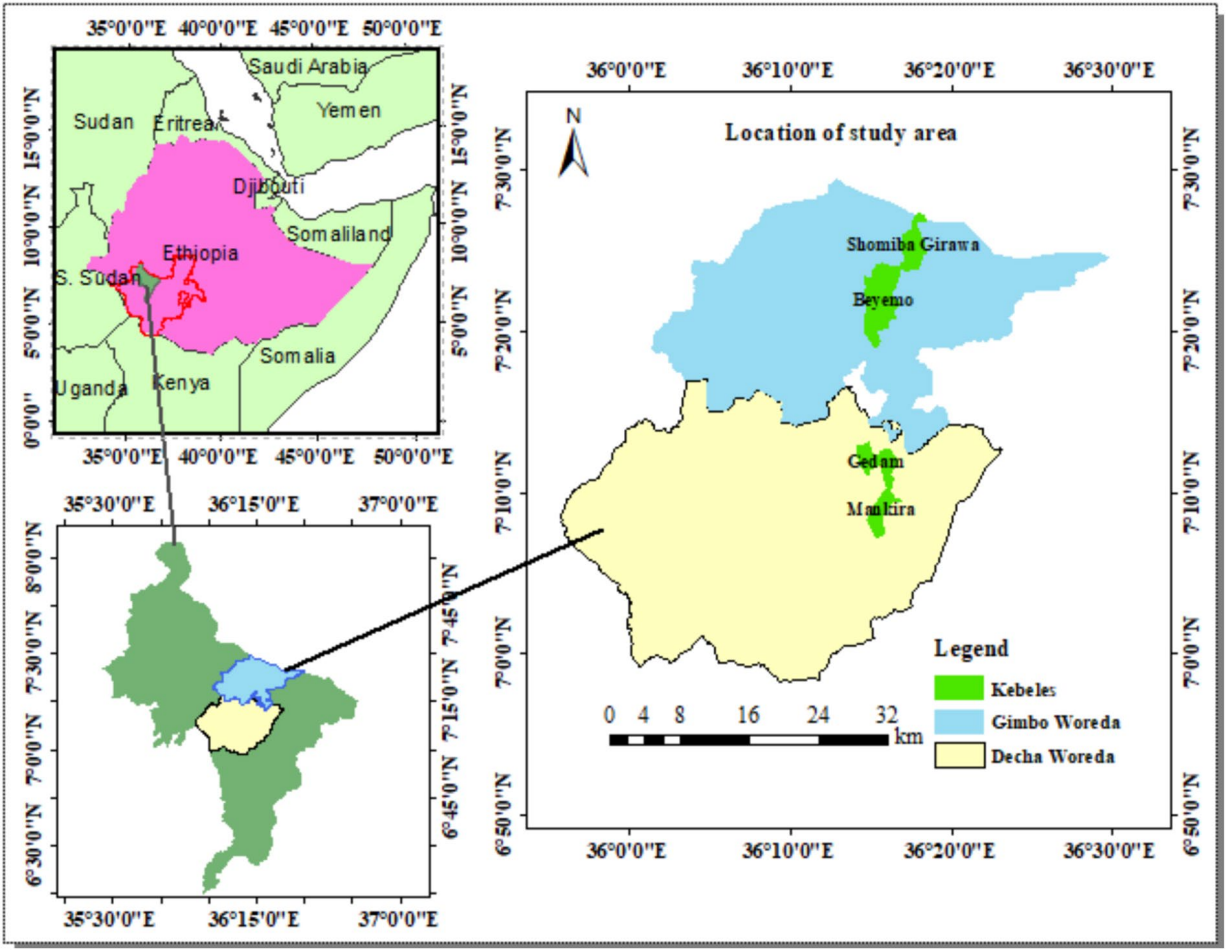
Map of the study area

The study focuses on the Decha and Gimbo districts, located within the Kafa Zone of South West Ethiopia Regional state (Fig. 1). Geographically, the area spans approximately 6°50′00″-7°30′00″ N latitude and 35°35′24″-36°30′00″ E longitude, situated about 460 km southwest of Addis Ababa^21^. The region is characterized by rugged highland topography with steep slopes, deep incised valleys, and elevations ranging from 894 to 2552 m a.s.l. The area supports an evergreen mountain forest at higher elevations to Afromontane moist evergreen broadleaf forests. It contributes to rivers such as Gojeb, Dincha, and Woshi within the catchment. It has 45,000 ha of wetlands draining into the Omo-Gibe and Baro-Akobo basins^21^.

Hydrologically, the area experiences a unimodal rainfall regime, with the majority of precipitation occurring during the main rainy season (Kiremt: June-September). The study area also receives relatively low rainfall from February to May. The area receives mean annual rainfall of about 1,800 mm, though recent decades have exhibited pronounced spatiotemporal variability in onset, duration, and intensity. The interaction between seasonal rainfall patterns, steep topography, and local soil hydraulic properties critically controls infiltration-runoff partitioning, which in turn dictates seasonal water availability, soil moisture retention, and agricultural drought vulnerability^17^.

The dominant soil types in the study area are Nitisols and Cambisols, characterized by moderate to high clay content, good internal drainage, and high base saturation. These soil properties influence water-holding capacity, erosion susceptibility during heavy rainfall events and crop root development. Ecologically, the region supports a transition from evergreen montane forests at higher elevations to Afromontane moist evergreen broadleaf forests at mid-elevations, providing a habitat for wild *Coffea arabica*. It was recognized as a UNESCO Biosphere Reserve in 2010, and as part of a global biodiversity hotspot contains approximately 45,000 ha of wetlands, with nearly half (47%) of the landscape under forest cover. It harbors rich biodiversity, including over 250 plant species, 300 mammal species, and 300 bird species, several of which are endemic or endangered^21^^,^^22^.

According to the 2020 Kafa Zone survey, the region has a population of 1,228,393 and 250,692 households. Over 90% of households rely on rain-fed subsistence agriculture and forest-based livelihoods. Coffee cultivation dominates the agricultural landscape, with approximately 10% grown under natural forest canopies and 65% in traditional home gardens. Other major crops include maize, teff, legumes, and enset, complemented by livestock production (cattle, poultry, and small ruminants). The region’s agroecological conditions, soil-water dynamics, and traditional farming systems maintain it as a vital in-situ gene bank for coffee genetic diversity^17^.

### Data sources and analysis

Historical daily rainfall and temperature data were obtained from 10 meteorological stations within the study area: Chira, Bitawoshi, Kemise, Wush Wush, Keresi, Sheda Tura, Goga Kemise, Bonga, Seka Chekorsa, Yebu (SM Table 7; SM Fig. 2). All station data were obtained from the Ethiopian Meteorological Institute (EMI).

**Fig. 2:**
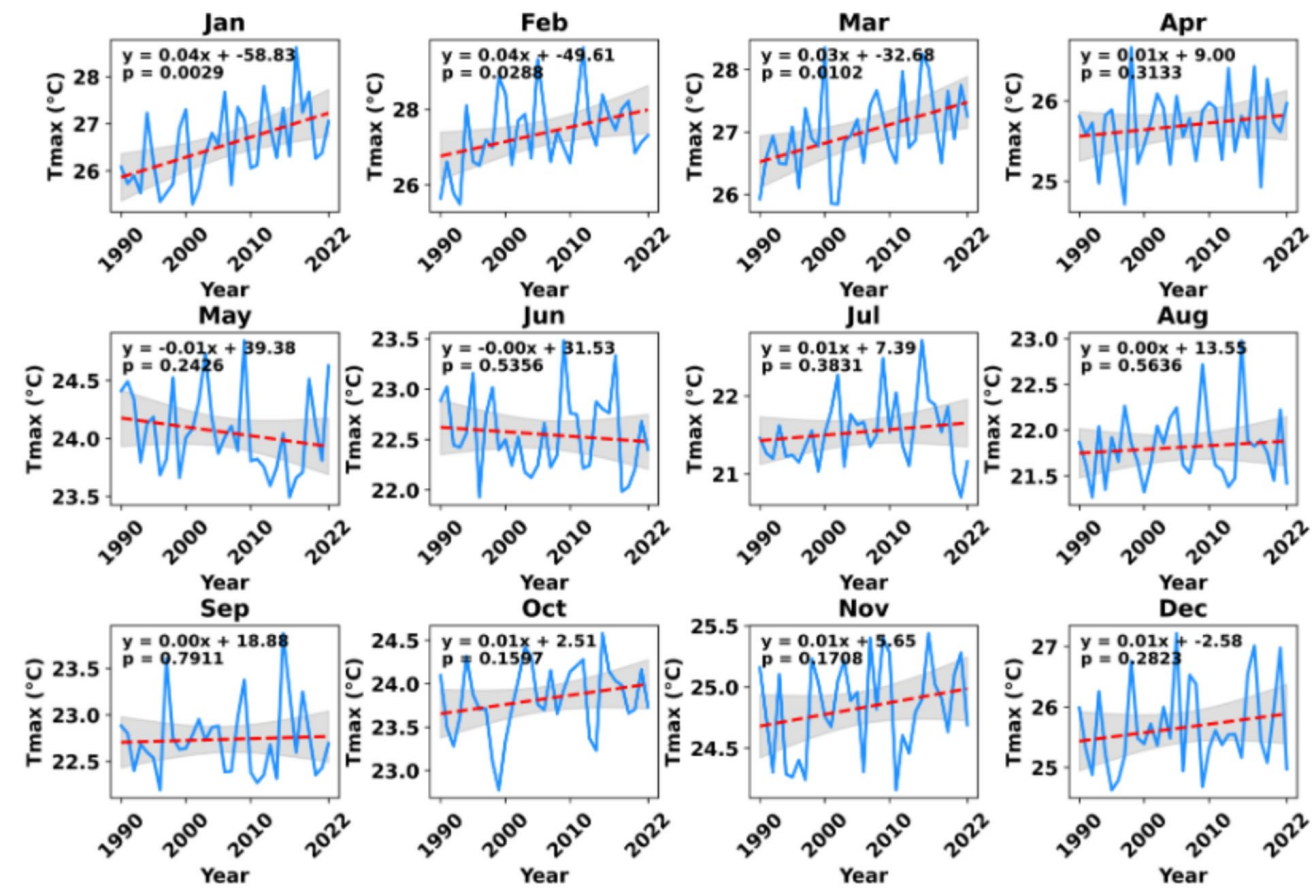
Monthly maximum temperature trends

#### Data screening, completeness, and homogenization

Prior to analysis, all station time series (1990-2022) underwent rigorous quality control. Data completeness was assessed for each station; records with >10% missing daily values in any month were flagged for gap-filling. Missing values were estimated using a regression-based approach leveraging correlations with neighboring reference stations (Pearson r > 0.7). Homogeneity of the time series was tested using the Standard Normal Homogeneity Test (SNHT) Alexandersson and the Buishand range test^23^ at the 95% confidence level. Stations exhibiting significant breakpoints were adjusted using the quantile-matching method relative to stable reference series. These procedures ensured that detected trends reflect climatic signals rather than non-climatic artifacts.

#### Gridded datasets and rationale for integration

In addition to observed station data, we integrated two high-resolution gridded products to ensure spatial completeness and support gap-filling: (i) precipitation from the Climate Hazards Group InfraRed Precipitation with Station data (CHIRPS) v2.0 (0.05° × 0.05°)^24^, and (ii) near-surface temperature from the ERA5-Land reanalysis (0.1° × 0.1°)^16^. CHIRPS was selected for its long-term consistency, satellite-gauge blending, and demonstrated performance across Ethiopia’s complex topography^24^. ERA5-Land was chosen for its physically based land-surface modeling and improved representation of temperature gradients in mountainous regions^25^. Station data were prioritized for local accuracy, while gridded datasets served three complementary purposes: (i) filling temporal gaps in station records, (ii) validating spatial patterns of observed trends, and (iii) enabling analysis in areas with sparse ground observations.

#### Spatial interpolation using regression kriging

To generate continuous spatiotemporal surfaces of rainfall and temperature, we applied regression kriging (RK) implemented in Python 3.12.4 using the scikit-gstat and pykrige libraries. RK was selected over deterministic methods (e.g., IDW) because it explicitly accounts for spatial autocorrelation in residuals and incorporates auxiliary covariates (elevation, slope, aspect) derived from a 30-m SRTM DEM. The method follows a two-step procedure: (1) a linear regression model predicts the climate variable using topographic covariates, and (2) ordinary kriging interpolates the regression residuals, which are then added to the regression predictions^26^. This hybrid approach is particularly effective in topographically complex regions, where elevation strongly modulates local climate.

#### Validation of gridded climate data

CHIRPS precipitation and ERA5-Land temperature datasets were validated against observations from ten meteorological stations in the Kafa Zone for 1990-2022. Gridded values were extracted at station coordinates using bilinear interpolation. Performance was evaluated at monthly, seasonal, and annual scales using RMSE, MAE, PBIAS, Pearson correlation (r), and Nash-Sutcliffe Efficiency (NSE).

To assess spatial robustness, leave-one-out cross-validation (LOOCV) was applied across stations^27^. Based on the validation results, station data were used for local trend analysis, while gridded datasets supported spatial interpolation and gap-filling. Regression kriging was employed to correct systematic biases and improve spatial accuracy (SM Table 8-11).

Validation against station observations indicated strong performance for both datasets. CHIRPS precipitation showed correlation coefficients ranging from r = 0.68-0.86, NSE = 0.58-0.79, and PBIAS between -5.3% and +7.1%. ERA5-Land temperature performed better, with r = 0.88-0.96, NSE = 0.80-0.92, and PBIAS ranging from -0.6 to +0.3 °C. Leave-One-Out Cross-Validation (LOOCV) of regression kriging further confirmed robust spatial predictive capacity, yielding R^2^ = 0.78 (RMSE = 142 mm yr⁻^1^) for precipitation and R^2^ = 0.94 (RMSE = 0.90 °C) for mean temperature. These results confirm the suitability of both datasets, following bias correction, for spatial interpolation and trend analysis in the study area (see SM Tables 8-11).

#### Trend detection and statistical analysis

To detect and quantify monotonic trends in rainfall and temperature time series, we applied the non-parametric Mann-Kendall (MK) test alongside Sen’s slope estimator. The MK test is distribution-free and robust to non-normality and outliers, making it well-suited for climate data^28^. Sen’s slope provides a robust estimate of trend magnitude (mm/year or °C/year). To address potential serial correlation, which can inflate MK test significance, we applied pre-whitening following the approach of Yue and Wang^29^ exclusively to the MK time series prior to trend testing. In contrast, the Innovative Trend Analysis (ITA) was conducted on the raw, unwhitened data, as its graphical and non-parametric framework inherently accommodates autocorrelation without requiring prior modification^30^. Additionally, data were aggregated to monthly, seasonal (*Kiremt*: June-September, *Belg*: February-May), and annual scales to assess trend consistency across temporal resolutions. Linear regression analysis was concurrently applied to illustrate trend direction and rate of change, facilitating comparison with prior studies that report parametric results. The combined use of non-parametric and parametric approaches, alongside rigorous pre-processing, ensures statistical robustness and methodological transparency.

#### Innovative trend analysis (ITA)

To complement the MK test and capture potential non-monotonic or hidden sub-trends, the ITA method proposed by Şen^31^ was applied. Unlike rank-based tests that assess only monotonic trends, ITA enables the quantitative detection of trends across low, medium, and high value ranges, thereby revealing partial or shifting patterns within a single time series. The trend slope is calculated as: ITA slope = (Ȳ - X̄) / *n*, where *n* is the number of observations in each half (total series length, *N* = 2*n*), and X̄ and Ȳ represent the means of the first and second sub-series, respectively^31^. ITA was selected for its capacity to detect non-monotonic behaviors (e.g., increasing extremes alongside stable means) that may be masked in traditional monotonic assessments, while its design allows direct application to original time series without serial correlation adjustments^30^.

### Mann-kendall test

The non-parametric Mann-Kendall test statistics ‘S’^32^^,^^33^ were employed to investigate rainfall and temperature trends using Equations 3 and 4

1 $$S=\sum_{k=1}^{n-1}\sum_{\mathrm{J}=\mathrm{k}+1}^{\mathrm{n}}\mathrm{s}\mathrm{g}\mathrm{n}\left({\mathrm{X}}_{\mathrm{j}}-{\mathrm{X}}_{\mathrm{k}}\right)$$

2 $$sgn\left({X}_{j}-{x}_{k}\right)=\left\{\begin{array}{c}1 if {X}_{j}-{X}_{k}>0 \\ 0 if {x}_{j}-{x}_{k}=0 \\ -1 if {x}_{j}-{x}_{k}<0\end{array}\right.$$

where *S* is the Mann-Kendall test statistic; ${X}_{j}$ and ${X}_{k}$ are the sequential data values of the annual time series, with *j>k*.

*The variance of S is:*

3 $$Var\left(S\right)=\frac{n\left(n-1\right)\left(2n+5\right)-{\sum}_{i=1}^{m}{t}_{i}\left({t}_{i}-1\right)\left({2t}_{i}+5\right)}{18}$$

where *n* is the number of data points, *m* is the number of tied groups, and ${t}_{i}$ is the number of data points in the *i* ^th^ tied group.

For *n>10*, the test statistic *S* is approximately normally distributed, and the standardized test statistic ${Z}_{s}$ is computed as follows:

4 $${\mathrm{Z}}_{\mathrm{s}}\left\{\begin{array}{cc}\frac{S-1}{\sqrt{Var(S)}}& \text{if }S>0\\ 0& \text{if }S=0\\ \frac{S+1}{\sqrt{Var(S)}}& \text{if }S<0\end{array}\right.$$

where *Var(S)* is defined in Equation 3. A positive ${Z}_{s}$ indicates an increasing trend, while a negative ${Z}_{s}$ indicates a decreasing trend^33^. For a significance level of *α =0.05*, the null hypothesis of no trend is rejected if $\mid {Z}_{s}\mid >1.96$.

### Sen’s slope estimator

The magnitude of the trend was estimated using Sen’s non-parametric method^34^. For a time, series of *n* annual values, the slope is calculated for all pairs of data points:

5 $${Q}_{i}=\frac{{X}_{j}-{X}_{k}}{j-k} {\mathrm{for}} i=\mathrm{1,2},\dots ,N;{\mathrm{and}} 1\le k<j\le n$$

where ${X}_{j}$ and ${X}_{k}$ are data values at times *j* and *k*, respectively. The number of slope estimates *N* is $\frac{n(n-1)}{2}$. Sen’s slope estimator (${Q}_{med}$)  is the median of all *N* values of ${{\boldsymbol{Q}}}_{{\boldsymbol{i}}}$. A positive Q_med indicates an increasing trend, and a negative value indicates a decreasing trend.

The slope magnitude is expressed in original units per year (e.g., mm year⁻^1^ for rainfall and °C year⁻^1^ for temperature), allowing direct interpretation of the rate of climatic change.

To assess the statistical reliability of the estimated slope, the 95% confidence interval for $\mathrm{Q}\mathrm{m}\mathrm{e}\mathrm{d}$ was computed using the non-parametric approach. If the confidence interval includes zero, the trend is considered not statistically significant at α = 0.05. This interval estimation complements the Mann-Kendall test and provides a robust measure of trend uncertainty, particularly for non-normally distributed or outlier-prone climate data.

When *Xj = Xk*, the corresponding *Qi* is undefined and excluded from the median calculation, following standard practice.

### Coefficient of variation (CV)

The Coefficient of Variation (CV) was calculated to quantify the relative interannual variability of rainfall, expressed as a percentage:

6 $$CV=\frac{\sigma }{\mu }x100$$

where σ is the standard deviation and μ is the long-term mean of annual rainfall over the reference period (1990-2022; n = 33 years). Following conventional classifications in regional climatology^35^ rainfall variability was categorized as shown in Table 1.

**Table 1:** Rainfall variability (CV) classes and agricultural implications

CV Range	Variability class	Agricultural interpretation
CV < 20%	Low	Relatively stable rainfall; favorable for rain-fed crop planning
20% ≤ CV ≤ 30%	Moderate	Noticeable interannual fluctuations; adaptive management recommended
CV > 30%	High/Strong	Highly unpredictable rainfall; elevated risk for crop failure and livelihood stress

### Standardized rainfall anomaly (SRA)

The Standardized Rainfall Anomaly (SRA), equivalent to a Z-score standardization was computed for the Kafa Zone at seasonal and annual timescales to identify wet and dry periods and assess meteorological drought frequency and severity. The SRA was calculated as:

7 $$SRAi=(Xi-\upmu )/\sigma$$

where Xi is the rainfall amount (seasonal, or annual) for year i, μ is the long-term mean rainfall over the reference period (1990-2022; n=33 years), and σ is the corresponding standard deviation. This approach follows established meteorological practice for anomaly detection and drought monitoring^36^.

#### Note on distributional assumptions

Since rainfall data are often positively skewed, we assessed normality using the Shapiro-Wilk test. For time series exhibiting significant skewness (p < 0.05), a log-transformation (X′ = ln (X+1)) was applied to the original data before calculating μ and σ for standardization, thereby improving the reliability of SRA-based drought classification^37^.

#### Drought severity classification

Drought events were categorized based on SRA thresholds calibrated to local agricultural impacts and aligned with percentile-based conventions used in Ethiopian climate studies, as shown in Table 2).

**Table 2:** SRA-based drought severity classification

SRA range	Drought class	Percentile
SRA ≤ -1.65	Extreme drought	≤ 5th
-1.65 < SRA ≤ -1.28	Severe drought	5^th^-10th
-1.28 < SRA ≤ -0.84	Moderate drought	10^th^-20th
SRA > -0.84	No drought / Near-normal	> 20th

## Results

### Maximum temperature trend

Mann-Kendall analysis indicated statistically significant increasing trends in maximum temperature for January (Zs = 2.88, p = 0.004, Sen’s slope = +0.04 °C year⁻^1^), February (Zs = 2.15, p = 0.031, Sen’s slope = +0.04 °C year⁻^1^), and March (Zs = 2.54, p = 0.011, Sen’s slope = +0.03 °C year⁻^1^), with the winter (Bega) seasonal aggregate also significant (Zs = 2.97, p = 0.003, Sen’s slope = +0.03 °C year⁻^1^). The annual trend was positive but not statistically significant (p = 0.055) (Fig. 2 and Table 3).

**Table 3:** Monthly MK trend test of temperature

Month	Tmin (°C)			Tmax (°C)			Tmean (°C)		
	Zs	p	Slope	Zs	p	Slope	Zs	p	Slope
Jan	-0.74	0.457	-0.01	2.88*	0.004	0.04	2.97*	0.003	0.02
Feb	0.52	0.598	0.00	2.15*	0.031	0.04	1.85	0.065	0.02
Mar	0.33	0.745	0.00	2.54*	0.011	0.03	3.48*	<0.001	0.03
Apr	1.78	0.075	0.01	1.07	0.285	0.01	3.45*	<0.001	0.02
May	1.79	0.074	0.01	-1.68	0.097	-0.01	3.29*	0.001	0.01
Jun	2.11*	0.035	0.01	-0.88	0.377	-0.01	2.54*	0.011	0.01
Jul	2.58*	0.010	0.01	1.04	0.299	0.01	2.97*	0.003	0.01
Aug	1.07	0.284	0.00	0.05	0.963	0.00	2.75*	0.006	0.01
Sep	0.44	0.675	0.00	-0.17	0.865	-0.00	2.97*	0.003	0.01
Oct	1.69	0.094	0.01	1.23	0.221	0.01	3.45*	<0.001	0.02
Nov	0.85	0.394	0.00	1.23	0.221	0.01	2.69*	0.008	0.01
Dec	0.02	0.988	0.00	1.04	0.299	0.01	2.97*	0.003	0.02
*Bega* (Oct-Jan)	-0.12	0.901	-0.00	2.97*	0.003	0.03	3.29*	0.001	0.02
*Belg* (Feb-May)	1.59	0.110	0.01	1.94	0.053	0.01	3.74*	<0.001	0.02
*Kiremt* (Jun-Sep)	2.41*	0.016	0.01	0.05	0.963	0.00	3.09*	0.002	0.01
*Annual*	1.88	0.060	0.01	1.92	0.055	0.02	3.52*	<0.001	0.02

*Indicates significant trend at P ≤ 0.05.

Innovative Trend Analysis (ITA) indicates a consistent increase in Tmax, with the highest warming in January (+0.79 °C) and winter (Bega) (+0.46 °C), while the lowest increase occurs during Kiremt (+0.13 °C). Overall, warming is more pronounced in the dry season (SM Table 1).

### Minimum temperature trend

**Fig. 3:**
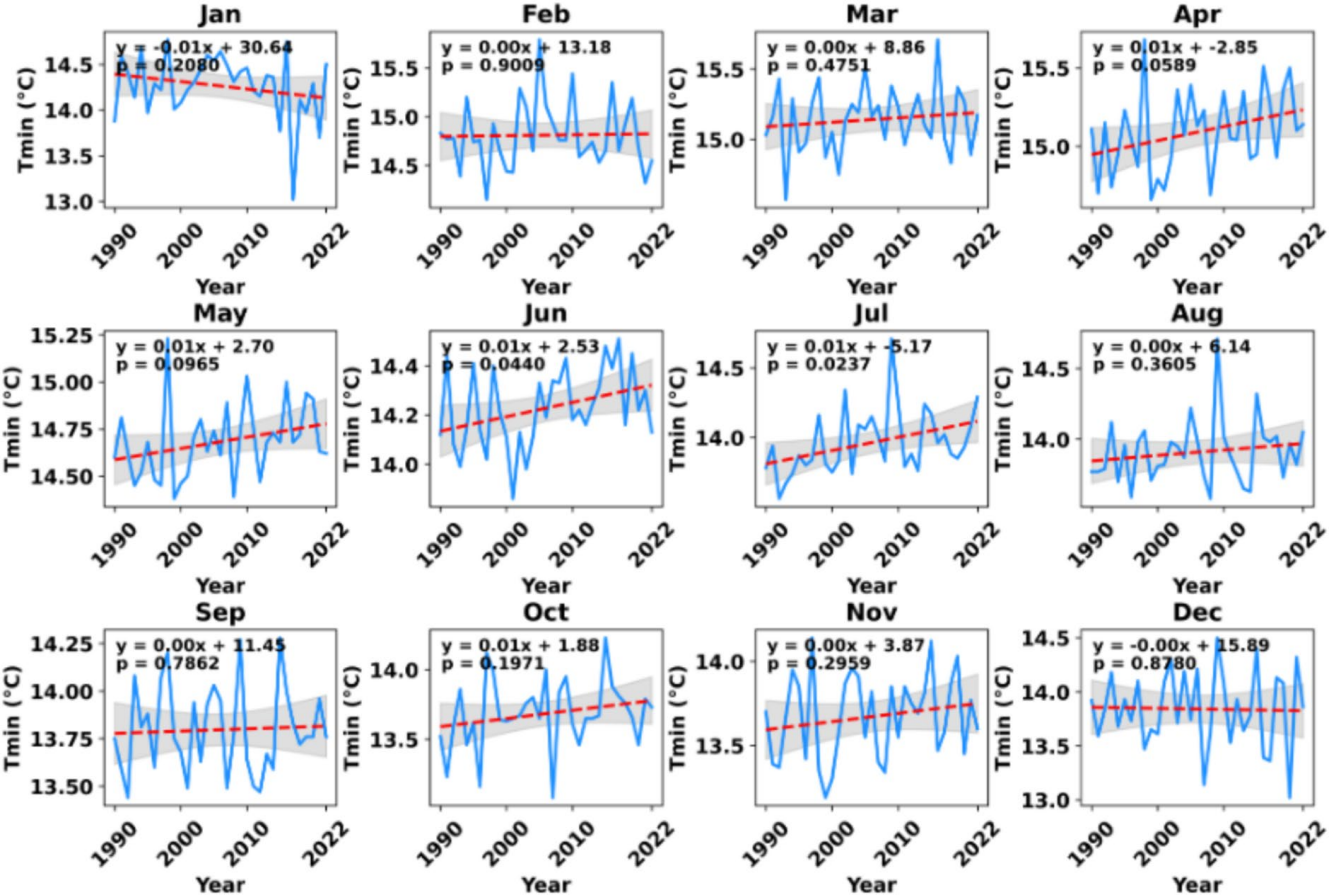
Monthly minimum temperature trends

Mann-Kendall analysis of monthly minimum temperature (Tmin) revealed statistically significant increasing trends in June (Zs = 2.11, p = 0.035, Sen’s slope = +0.01 °C year⁻^1^) and July (Zs = 2.58, p = 0.010, Sen’s slope = +0.01 °C year⁻^1^), with the Kiremt seasonal aggregate also significant (Zs = 2.41, p = 0.016, Sen’s slope = +0.01 °C year⁻^1^). The annual trend was positive but not statistically significant (p = 0.060) (Figure 3).

Innovative Trend Analysis (ITA) shows a consistent increase in Tmin, with the highest warming in July (+0.76 °C) and June (+0.69 °C), while the lowest increase occurs in January, September, and December (+0.33 °C). Seasonally, warming is strongest during summer (Kiremt) (+0.55 °C) and spring (Belg) (+0.50 °C), indicating more pronounced nighttime warming in the wet seasons (SM Table 2).

#### Temporal variability and trends of temperature

### Mean temperature trend

**Fig. 4:**
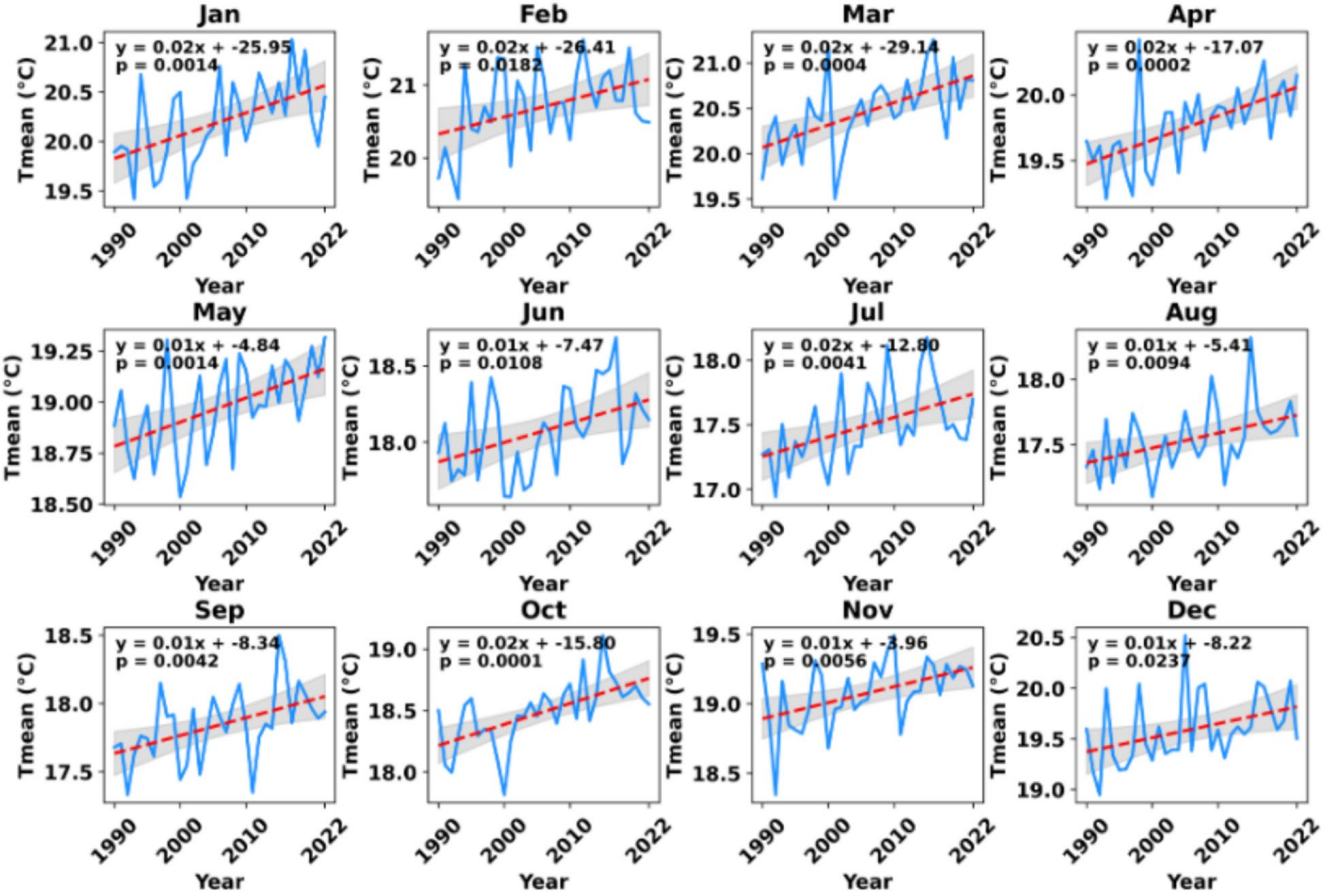
Time series trends in monthly mean temperature

Mann-Kendall analysis revealed statistically significant increasing trends in mean temperature (Tmean) across multiple temporal scales. Eleven out of twelve months exhibited significant trends (0.001 ≤ p ≤ 0.011), with Sen’s slopes ranging from +0.01 to +0.03 °C year⁻^1^. All seasonal aggregates were highly significant (p ≤ 0.001), and the annual trend was also strongly significant (Zs = 3.52, p < 0.001, Sen’s slope = +0.02 °C year⁻^1^) *(*Table 3 and Fig. 4).

### Station-based temperature trend and spatial distribution

Mann-Kendall analysis of station-level temperature trends indicates that statistically significant increases in mean maximum temperature (Tmax) were observed at Keresi (Zs = 2.81, p = 0.005, Sen’s slope = +0.012 °C year^−1^) and Yebu (Zs = 2.69, p = 0.007, Sen’s slope = +0.011 °C year^−1^), while the remaining stations showed non-significant trends with slopes ranging from -0.008 to +0.008 °C year^−1^.

For mean minimum temperature (Tmin), significant increasing trends were detected at most stations, including Chira (Zs = 2.88, p = 0.004), Kemise (Zs = 1.98, p = 0.048), Keresi (Zs = 2.97, p = 0.003), Sheda Tura (Zs = 2.15, p = 0.032), Goga Kemise (Zs = 2.88, p = 0.004), Bonga (Zs = 2.31, p = 0.021), Seka Chekorsa (Zs = 2.06, p = 0.039), and Yebu (Zs = 3.48, p < 0.001), with Sen’s slopes ranging from +0.009 to +0.016 °C year^−1^. Bitawoshi and Wush Wush exhibited non-significant trends.

Mean temperature (Tmean) showed statistically significant increasing trends at all stations except Bitawoshi and Wush Wush, with Zs values ranging from 2.48 to 3.59 (p ≤ 0.013) and Sen’s slopes between +0.009 and +0.015 °C year^−1^ (Table 4).

**Table 4:** Trends in mean, maximum, and minimum temperature (1990-2022)

Station	Mean maximum temperature (°C)			Mean minimum temperature (°C)			Mean temperature (°C)		
	Zs	p	Slope	Zs	p	Slope	Zs	p	Slope
Chira	0.94	0.345	0.002	2.88*	0.004	0.012	3.37*	<0.001	0.011
Bitawoshi	-0.87	0.384	-0.006	1.52	0.129	0.004	0.95	0.342	0.003
Kemise	-0.45	0.653	-0.003	1.98*	0.048	0.009	1.67	0.095	0.006
Wush Wush	-1.12	0.263	-0.008	1.21	0.226	0.004	0.73	0.465	0.003
Keresi	2.81*	0.005	0.012	2.97*	0.003	0.013	3.54*	<0.001	0.013
Sheda Tura	1.15	0.250	0.005	2.15*	0.032	0.010	3.45*	<0.001	0.012
Goga Kemise	1.64	0.101	0.008	2.88*	0.004	0.016	3.12*	0.002	0.011
Bonga	0.72	0.471	0.004	2.31*	0.021	0.011	2.95*	0.003	0.010
Seka Chekorsa	0.89	0.374	0.004	2.06*	0.039	0.010	2.48*	0.013	0.009
Yebu	2.69*	0.007	0.011	3.48*	<0.001	0.014	3.59*	<0.001	0.015

*Indicates significant trend at P ≤ 0.05.

**Fig. 5:**
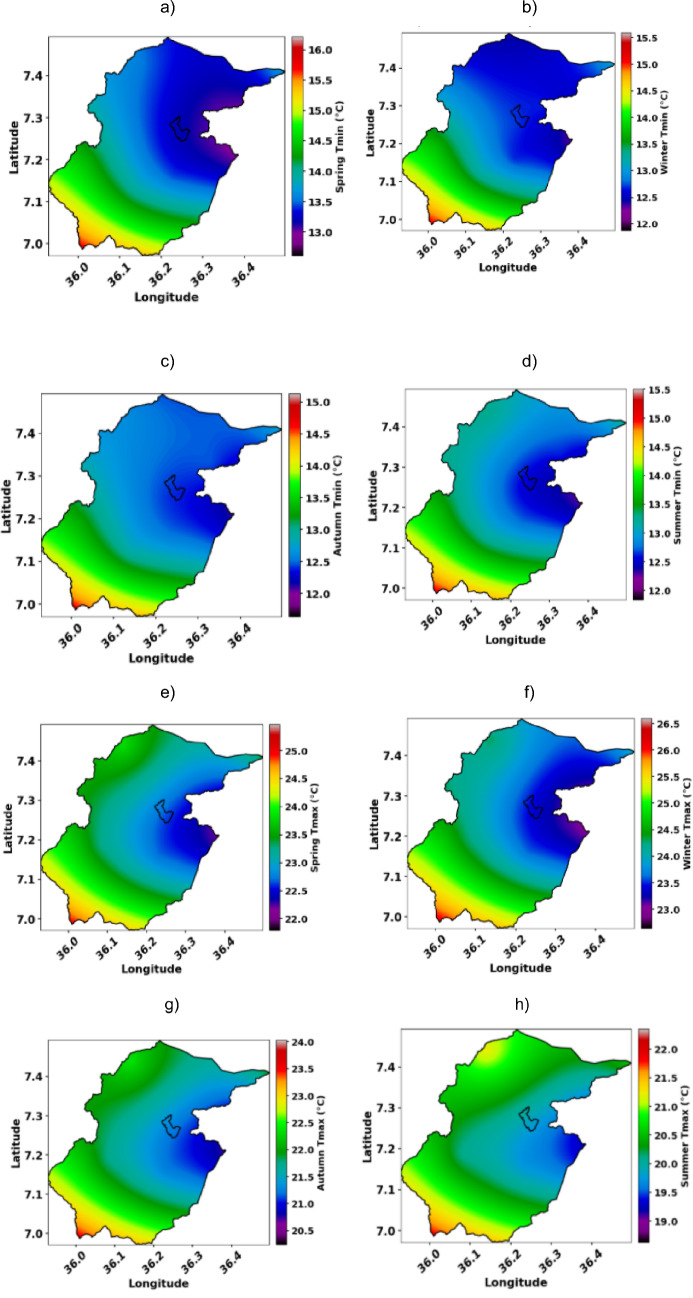
Spatial distribution of seasonal temperatures (1990-2022): spring minimum (**a**), winter minimum (**b**), autumn minimum (**c**), summer minimum (**d**), spring maximum (**e**), winter maximum (**f**), autumn maximum (**g**), and summer maximum (**h**)

With respect to spatial distributions, seasonal temperatures show distinct regional patterns. In nearly all seasons, the southern part of the study area experiences higher temperatures, while the northern and eastern regions remain cooler. This pattern is consistent for both maximum (Tmax) and minimum (Tmin) temperatures across the seasons (Fig. 5).

#### Annual and seasonal temperature trend (°C)

Annual and seasonal temperatures analyses indicate consistent increasing trends across Tmin, Tmean, and Tmax. Minimum temperature (Tmin) shows an upward trend, with slopes ranging from +0.009 to +0.013 °C year⁻^1^ (p ≤ 0.0033). Mean temperature (Tmean) exhibits strong and highly significant increases across all seasons (p ≤ 0.0003), with slopes between +0.013 and +0.020 °C year⁻^1^. In contrast, maximum temperature (Tmax) displays weaker and more variable patterns, with a statistically significant increase observed only during winter (slope = +0.032 °C year⁻^1^, p = 0.0019), while other seasons remain non-significant (p > 0.05).

**Fig. 6:**
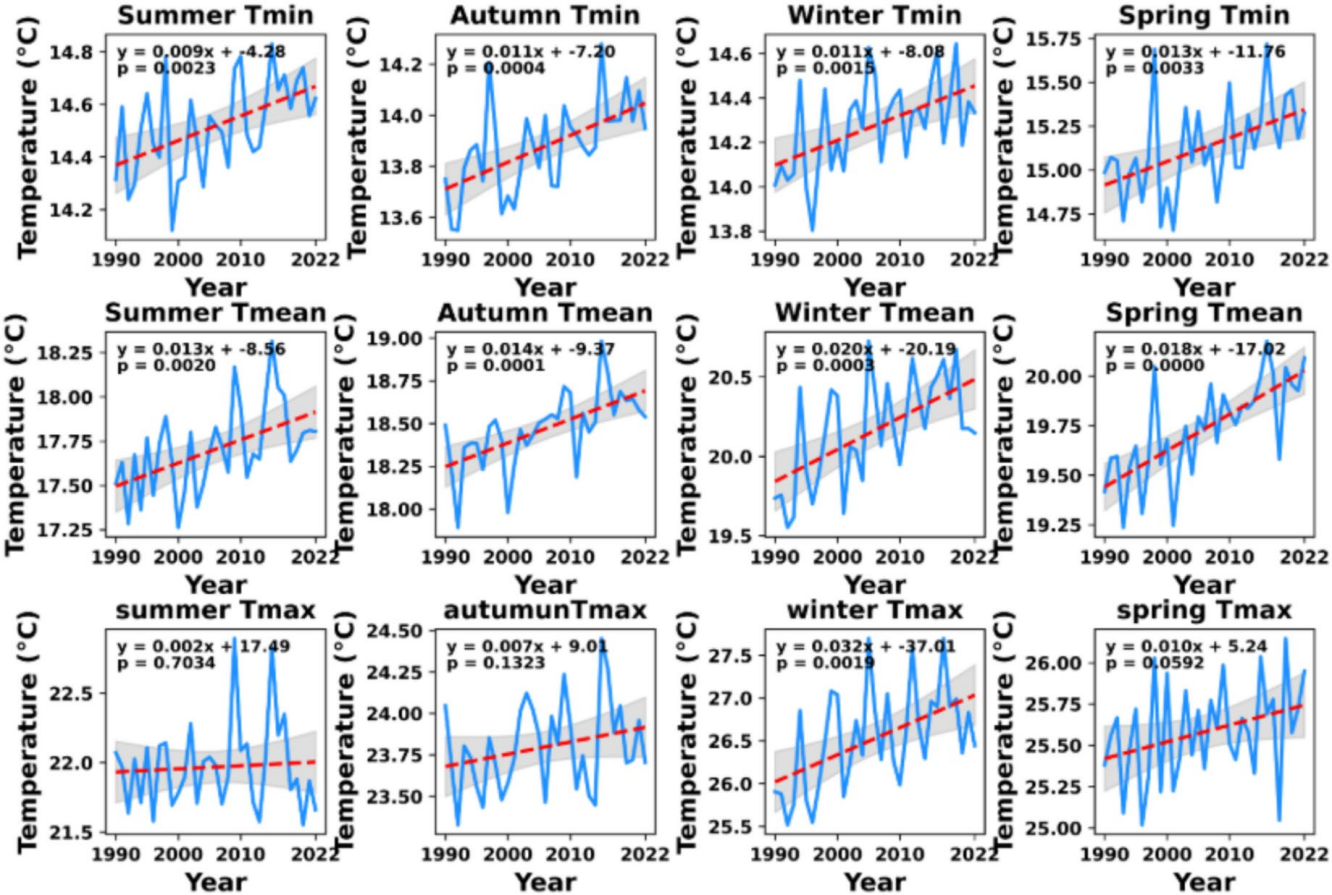
Seasonal trends of minimum, mean and maximum temperatures

**Fig. 7:**
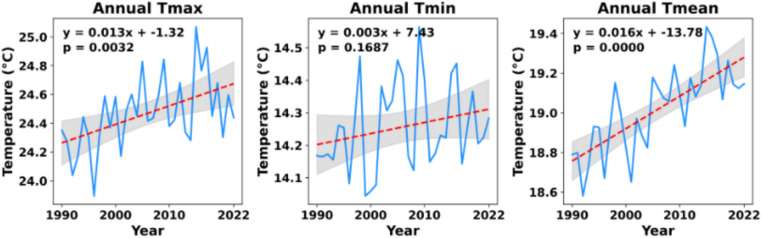
Time series trends in monthly and annual mean temperatures

At the annual scale, Tmax and Tmean show statistically significant increasing trends (p = 0.0032 and p < 0.001, respectively), whereas Tmin exhibits a positive but non-significant trend (p = 0.1687) (Figs. 6 and 7).

### Temperature anomalies (1990-2022)

**Fig. 8:**
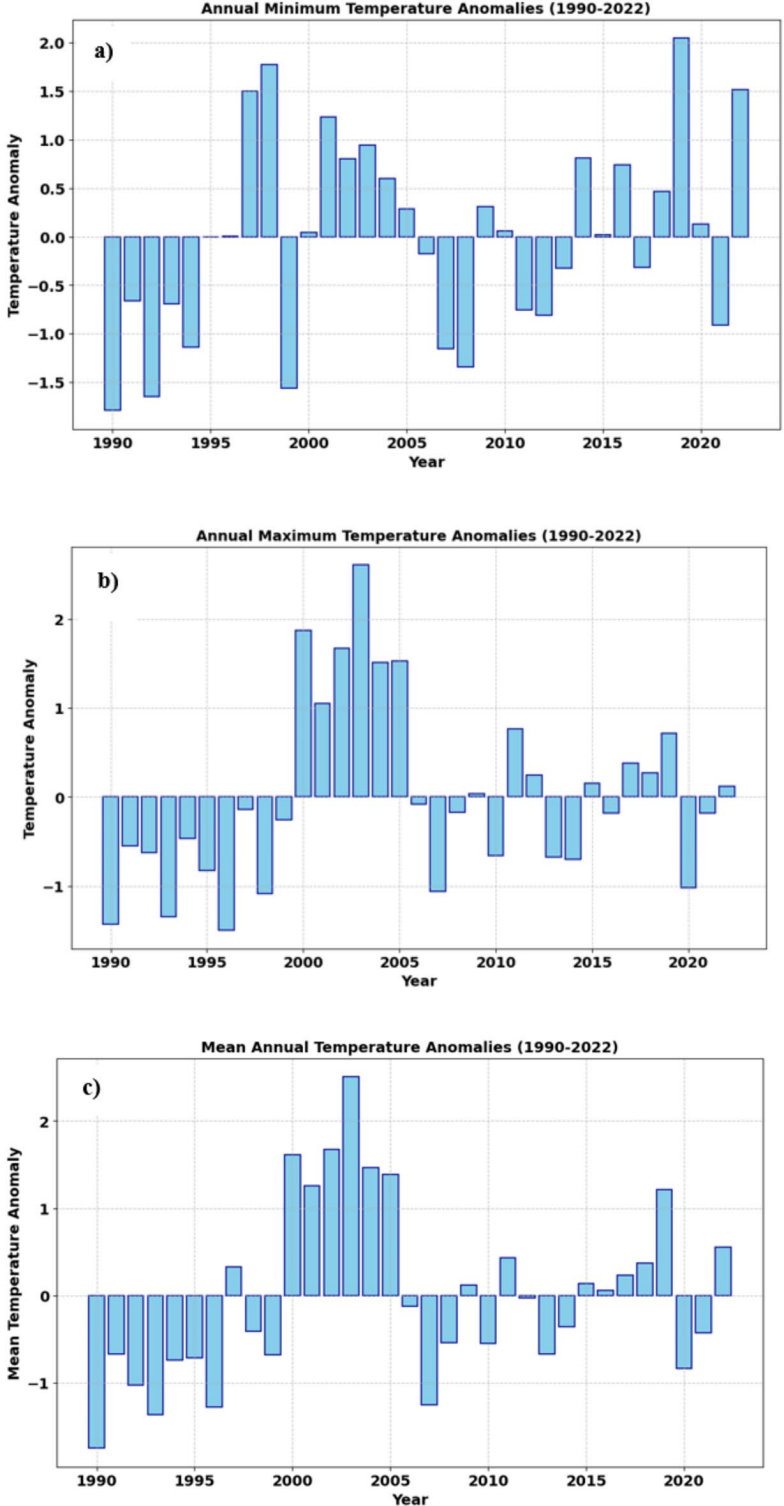
Standardized anomalies of minimum (**a**), maximum (**b**), and annual mean (**c**) temperatures

Annual temperature anomalies (1990-2022) exhibited metric-specific variability. Tmin anomalies ranged from -1.70 °C (1990) to +2.1 °C (2019), with predominantly negative values during the 1990s and mostly positive anomalies after2000. Tmax anomalies varied from -1.75 °C (1996) to +2.75 °C (2003), showing decadal-scale sign reversals without sustained trend. Tmean anomalies shifted from predominantly negative values in the 1990s to positive values during 2000-2005 (peak: +2.6 °C in 2003), followed by fluctuations that remained largely positive (Fig. 8).

### Seasonal trends of maximum and minimum temperature (1990-2022)

Seasonal analysis reveals a consistent warming pattern in both maximum and minimum temperatures, though with varying magnitude and spatial significance. Maximum temperature shows stronger and more widespread increases, particularly during winter and spring, while minimum temperature exhibits a moderate but steady rise. The warming rates generally range from 0.01 to 0.05 °C/year, indicating gradual but persistent change. However, localized non-significant trends at some stations highlight the influence of topography, forest cover, and moisture conditions, suggesting that the thermal response across the Kafa Biosphere Reserve is spatially heterogeneous but directionally consistent toward warming (SM Table 6-7).

### Rainfall data analysis: monthly, seasonal, and annual trends

#### Monthly and annual rainfall patterns

**Fig. 9:**
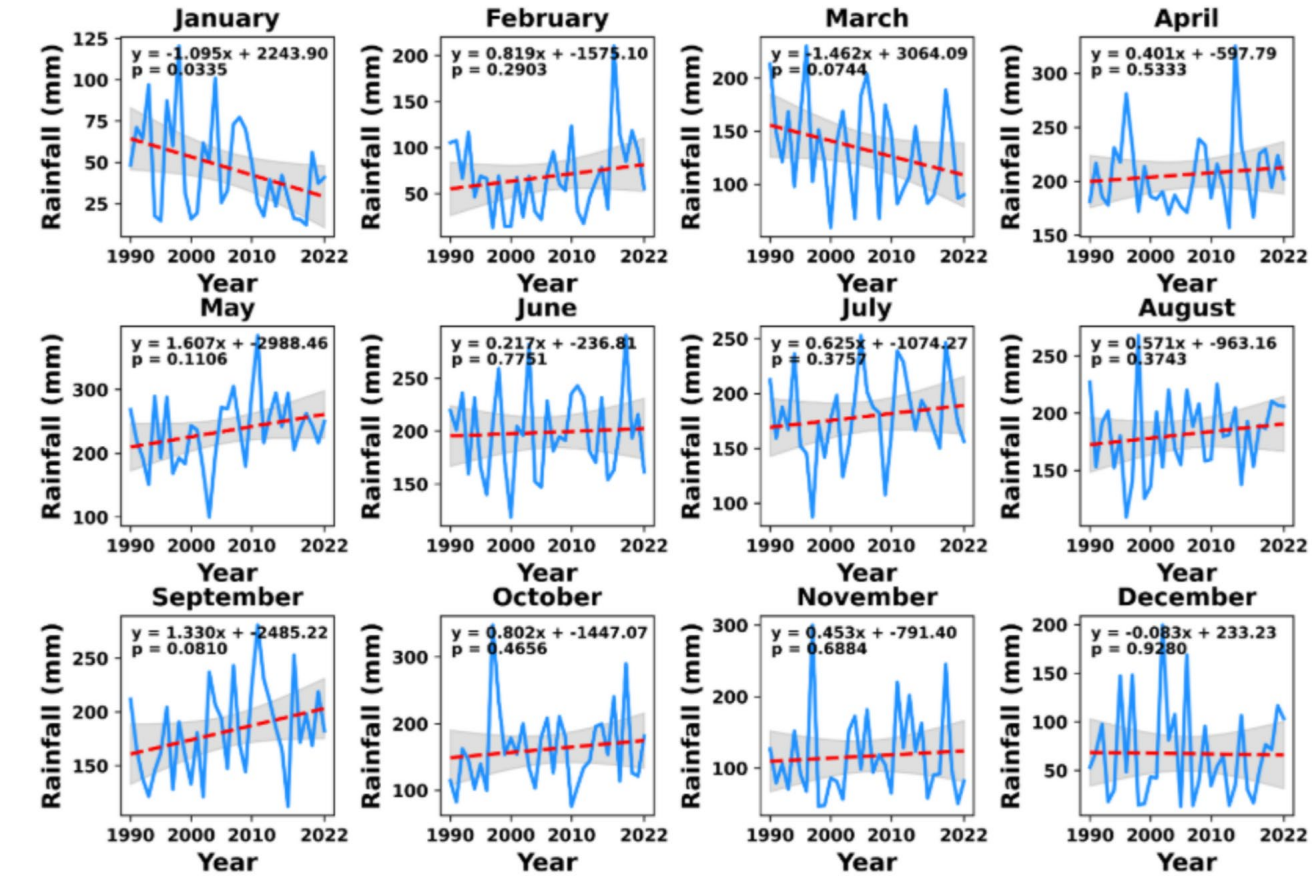
Monthly rainfall trends

The analysis of monthly rainfall reveals noticeable seasonal variability. The wettest months are May (235 mm) and April (206 mm), contributing the largest proportions to the annual total, while the driest months are January (47 mm) and December (67 mm). Rainfall exhibits high interannual variability, particularly during the short rainy and dry months, with the coefficient of variation ranging from moderate to very high values. A statistically significant increasing trend is observed in September, reflecting intensifying rainfall late in the main rainy season. Annual total rainfall averages 1776 mm, showing a significant upward trend over the study period, indicating an overall increase in annual precipitation despite monthly fluctuations (Table 5 and Fig. 9).

**Table 5:** Basic statistics, Mann-Kendall trend, Sen’s slope of rainfall

Month	Min (mm)	Max (mm)	Mean (mm)	% of Annual	SD (mm)	CV (%)	Zs	p-value	Sen’s Slope (mm/yr)
Jan	12.19	120.34	46.80	2.64	28.54	60.99	-0.74	0.457	-0.12
Feb	13.22	210.18	68.34	3.85	41.76	61.10	0.33	0.745	0.05
Mar	59.33	230.10	132.20	7.44	44.89	33.96	-0.74	0.457	-0.18
Apr	157.10	325.23	206.17	11.61	34.47	16.72	0.44	0.675	0.08
May	100.09	384.66	235.42	13.26	54.92	23.33	0.74	0.457	0.21
Jun	118.92	290.27	198.85	11.20	40.63	20.43	0.02	0.988	0.00
Jul	87.45	252.81	179.18	10.09	37.91	21.16	0.44	0.675	0.09
Aug	109.23	267.77	181.52	10.22	34.52	19.02	0.74	0.457	0.15
Sep	111.93	281.01	181.94	10.24	41.72	22.93	2.15*	0.031	1.45
Oct	75.86	347.81	161.65	9.10	58.95	36.47	0.52	0.603	0.23
Nov	46.47	300.14	116.80	6.58	60.37	51.69	0.33	0.745	0.11
Dec	12.93	199.48	67.20	3.78	48.91	72.78	0.12	0.901	0.04
Annual	1378.99	2310.71	1776.08	100.00	186.30	10.49	2.41*	0.016	2.69

*indicates statistically significant trend at p < 0.05 (Mann-Kendall test). CV: coefficient of variation (%) = (SD/Mean) × 100. %: monthly contribution to annual rainfall. Sen’s slope: magnitude of trend (mm/year).

### Precipitation concentration index (PCI) analysis

The PCI analysis indicates that annual rainfall is moderately concentrated, with 60.6 % falling in the 10-15 PCI range and a mean annual PCI of 12.3 ± 1.8. Seasonal analysis reveals a much stronger concentration during both Kiremt (June-September) and Belg (February-May) rains, with 75.8 % and 61.8 %, respectively, exhibiting very high concentration (PCI > 20), and mean PCI values of 24.7 ± 3.1 for Kiremt and 23.9 ± 4.2 for Belg. These results highlight that while annual rainfall is relatively evenly distributed, seasonal rainfall is highly concentrated within the specific rainy seasons (Table 6).

**Table 6:** Annual, summer and spring rainfall concentration Index (PCI) for 1990-2022

PCI Class	Interpretation	Annual rainfall (% of years)	*Kiremt* (Jun-Sep) (% of years)	*Belg* (Feb-May) (% of years)
< 10	Uniform distribution	39.4	-	-
10-15	Moderate concentration	60.6	-	-
16-20	High concentration / irregular	-	24.2	18.2
> 20	Very high concentration / strongly seasonal	-	75.8	61.8
Mean PCI ± SD		12.3 ± 1.8	24.7 ± 3.1	23.9 ± 4.2

### Station-level rainfall concentration

Analysis of the Precipitation Concentration Index (PCI) across individual stations indicate that rainfall is predominantly moderately concentrated (PCI 10-15) throughout the study area. Mean PCI values range from 10.3 ± 0.6 at Sheda Tura and Goga Kemise to 11.6 ± 0.9 at Yebu, with the regional mean at 10.7 ± 0.8. Uniform rainfall (PCI < 10) occurs infrequently, accounting for 9.4 % of station, while high concentration (PCI 15-20) is rare (3.0 %), and very high concentration (PCI ≥ 20) is almost absent (0.3 %). These results demonstrate that, at the station level, rainfall in the region is largely moderate in temporal distribution, with only occasional departures toward uniformity or high concentration (Table 7).

**Table 7:** Rainfall concentration index (PCI) range and rainfall categories (1990-2022).

Station	PCI range	Mean PCI ± SD	Uniform (PCI < 10)	Moderate (10 ≤ PCI < 15)	High (15 ≤ PCI < 20)	Very High (PCI ≥ 20)
Chira	9.7-13.8	11.4 ± 1.1	3 (9.1%)	28 (84.8%)	2 (6.1%)	0 (0.0%)
Bitawoshi	9.5-11.7	10.6 ± 0.7	11 (33.3%)	21 (63.6%)	1 (3.0%)	0 (0.0%)
Kemise	9.4-11.7	10.5 ± 0.7	13 (39.4%)	19 (57.6%)	1 (3.0%)	0 (0.0%)
Wush Wush	9.4-11.7	10.5 ± 0.7	13 (39.4%)	19 (57.6%)	1 (3.0%)	0 (0.0%)
Keresi	9.4-11.7	10.6 ± 0.8	11 (33.3%)	21 (63.6%)	1 (3.0%)	0 (0.0%)
Sheda Tura	9.3-11.4	10.3 ± 0.6	10 (30.3%)	22 (66.7%)	1 (3.0%)	0 (0.0%)
Goga Kemise	9.3-11.4	10.3 ± 0.7	10 (30.3%)	22 (66.7%)	1 (3.0%)	0 (0.0%)
Bonga	9.0-12.7	10.8 ± 0.9	14 (42.4%)	18 (54.5%)	1 (3.0%)	0 (0.0%)
Seka Chekorsa	9.4-11.7	10.5 ± 0.7	8 (24.2%)	24 (72.7%)	1 (3.0%)	0 (0.0%)
Yebu	10.0-13.2	11.6 ± 0.9	1 (3.0%)	30 (90.9%)	2 (6.1%)	0 (0.0%)
Regional Mean	9.0-13.8	10.7 ± 0.8	9.4%	87.3%	3.0%	0.3%

### Inter-annual rainfall variability and implications for coffee farming

**Fig. 10:**
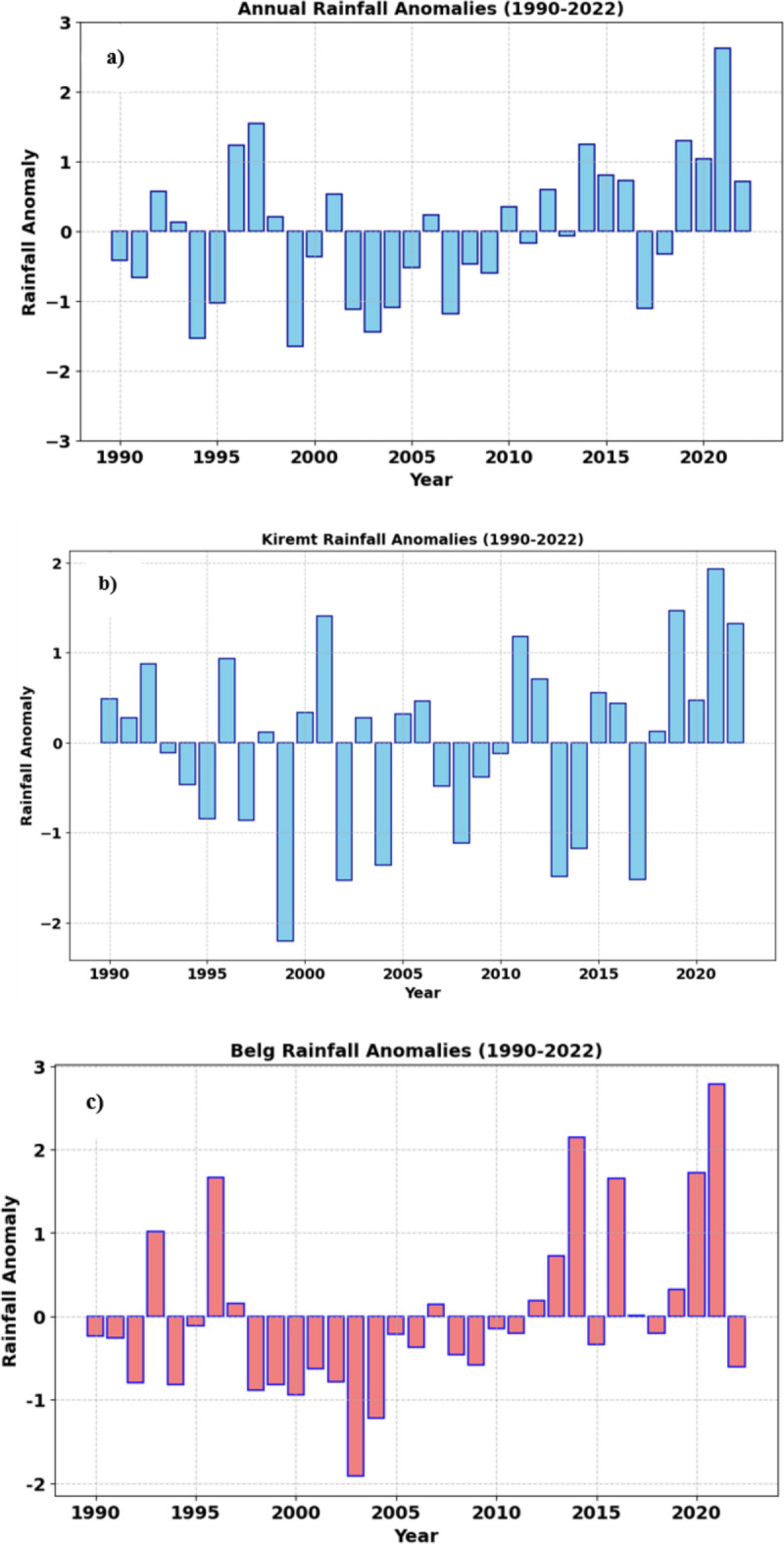
Standardized anomalies of annual (**a**), Kiremt (summer) (**b**), and belg (spring) (**c**), rainfall

Rainfall in the study area exhibited pronounced inter-annual variability from 1990 to 2022, without a clear long-term trend (Fig. 10). Sharp oscillations are evident between severe droughts such as in 1999, and intense wet periods, such as in 2021, across annual, Kiremt (June-September), and Belg (February-May) seasons. This erratic see-saw pattern, with substantial deficits closely followed by surpluses, highlights a climate regime characterized by increasing unpredictability. For coffee farming households, such variability poses a greater challenge than a gradual trend, disrupting the timing and reliability of growing seasons and intensifying the risks of both water stress and flood-related damage within short periods (Fig. 10).

### Seasonal and annual rainfall distribution across stations

**Fig. 11:**
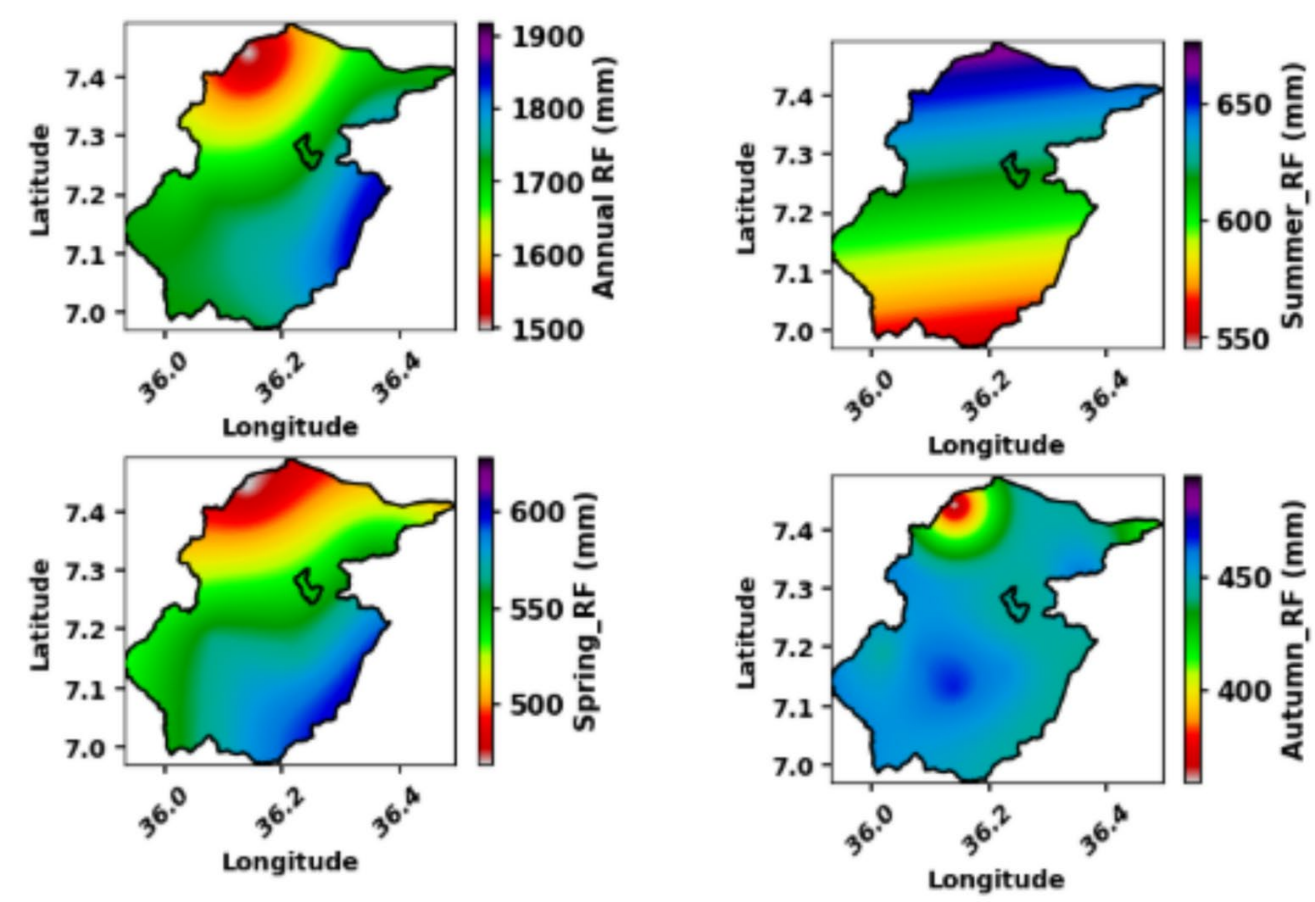
Spatial patterns of annual (**a**), spring (**b**), summer (**c**), and autumn (**d**) rainfall over a 33-year period

**Fig. 12:**
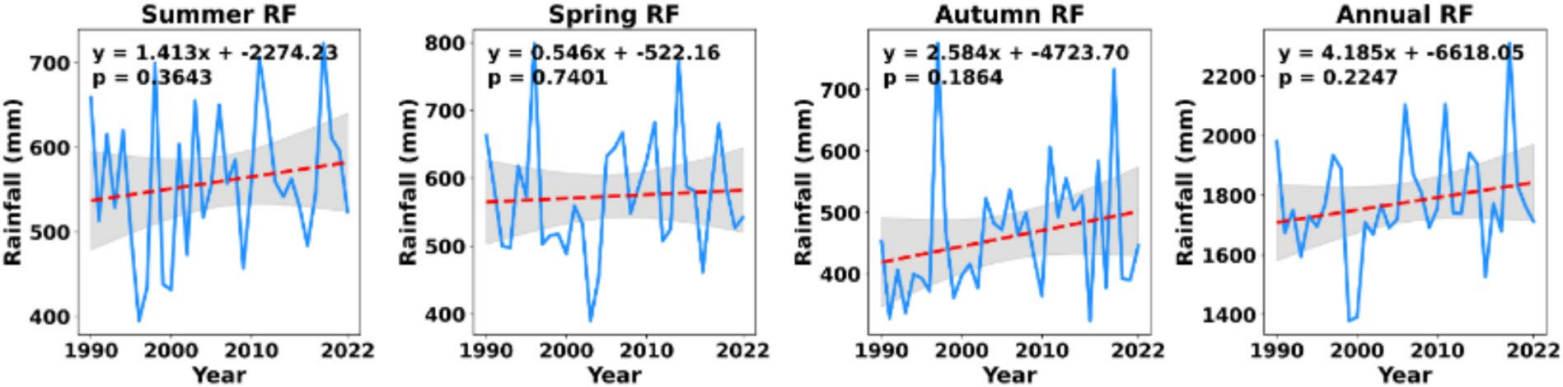
Temporal trends of annual, summer, autumn and spring rainfall over 33 years

Rainfall distribution across the study area exhibits clear seasonal patterns and moderate spatial variability. During the Belg season (February-May), mean rainfall ranges from 352.5 mm at Yebu to 585.1 mm at Keresi, contributing approximately 21-35 % of the annual total, with coefficients of variation (CV) between 15-23 %, indicating moderate interannual variability. The Kiremt season (June-September) is wetter, with mean ranging from 449.7 mm at Chira to 655.8 mm at Yebu, accounting for 31-39 % of annual rainfall, and CV values of 12-19 %, reflecting relatively stable seasonal precipitation compared to Belg. Annual totals range from 1,255.8 mm at Chira to 1,817.4 mm at Seka Chekorsa with contributions consistently summing to 100 % and CV values mostly between 10-12 %, except at Bonga (17.4 %), indicating moderate interannual variability across the region. Trend analysis suggests slight positive or negative slopes at some stations, but these are not statistically significant, underscoring that rainfall variability, rather than long-term directional change, dominates the hydroclimatic regime (Table 8 and Figs. 11-12).

**Table 8:** Spatial variability and trends of mean annual, summer, and spring rainfall

Season	Attribute	Chira	Bitawoshi	Kemise	Wush Wush	Keresi	Sheda Tura	Goga Kemise	Bonga	Seka Chekorsa	Yebu
Belg (February- May)	Mean (mm)	434.0	552.0	573.8	577.9	585.1	565.6	543.4	538.4	566.0	352.5
	Contribution (%)	34.6	32.7	32.3	32.3	32.3	32.0	31.5	31.2	31.1	21.0
	CV (%)	18.9	15.3	15.3	15.6	17.3	17.7	15.1	19.5	15.7	23.4
	Zs	-0.05	0.07	0.08	0.12	0.08	0.08	0.03	0.13	0.11	-0.04
	*p*	0.96	0.94	0.94	0.91	0.94	0.94	0.98	0.90	0.91	0.97
	Slope	-0.03	0.05	0.06	0.09	0.06	0.06	0.02	0.10	0.08	-0.03
Kiremt (Jun - September)	Mean (mm)	449.7	536.3	559.6	559.5	566.6	557.3	570.0	588.1	622.5	655.8
	Contribution (%)	35.8	31.8	31.5	31.3	31.3	31.5	33.1	34.1	34.3	39.0
	CV (%)	18.6	14.8	15.0	14.7	15.8	15.2	13.8	14.4	12.4	13.1
	Zs	0.10	0.08	0.10	0.10	0.09	0.08	0.00	0.14	0.05	0.08
	*p*	0.92	0.94	0.92	0.92	0.93	0.94	1.00	0.89	0.96	0.94
	Slope	0.08	0.06	0.08	0.08	0.07	0.06	0.00	0.11	0.04	0.06
Annual	Mean (mm)	1,255.8	1,688.6	1,776.1	1,788.7	1,811.8	1,769.9	1,723.7	1,726.7	1,817.4	1,681.4
	Contribution (%)	100	100	100	100	100	100	100	100	100	100
	CV (%)	12.4	10.6	10.5	11.1	11.8	11.8	10.0	17.4	10.6	10.5
	Zs	-0.05	0.10	0.14	0.19	0.22	0.20	0.16	0.27	0.17	0.08
	*p*	0.96	0.92	0.89	0.85	0.83	0.84	0.87	0.79	0.86	0.94
	Slope	-0.15	0.32	0.45	0.61	0.71	0.64	0.51	0.87	0.55	0.26

Asterisks denote statistical significance levels: *p ≤ 0.05.

## Discussions

The climatic trajectories documented across the Gimbo and Decha districts between 1990 and 2022 point toward a gradual transformation of the local environmental baseline, characterized by discernible warming, shifting precipitation dynamics, and heightened interannual variability. These observed patterns situate the Kafa Zone within broader regional assessments that document accelerated thermal increases and hydroclimatic instability across the Ethiopian highlands^4, 7^. For Coffea arabica, a perennial crop recognized for its relatively narrow bioclimatic envelope, such deviations from historical norms may carry meaningful implications for phenological synchronization, physiological performance, and yield stability. The crop’s established sensitivity to temperatures outside the 18-22°C optimum, coupled with its reliance on well-distributed seasonal moisture, suggests that even moderate climatic shifts could alter the suitability of current cultivation zones over time^38^,^39^. The convergence of rising thermal metrics and increasingly erratic rainfall distribution appears to be redefining the agroecological context in which smallholder coffee systems operate, necessitating a closer examination of how these trends intersect with crop-specific thresholds and local management practices.

Temperature trends within the study area show a consistent warming signal, though the magnitude and statistical significance vary across temporal scales and thermal metrics. Mean temperature (Tmean) demonstrated the most robust and statistically significant upward tendencies across nearly all months and seasons (annual slope: +0.02 °C year⁻^1^, p < 0.001), while maximum and minimum temperatures exhibited more seasonally heterogeneous patterns, this aligns with projections of sustained warming across eastern Africa^6, 7^. Maximum temperatures show statistically significant increases during the early dry months (January-March) season, while minimum temperatures exhibit notable upward shifts during the core rainy period (June**-**July) season. This seasonal divergence in thermal trends warming Tmax in the dry season and warming Tmin in the rainy season could potentially indicate a shift in diurnal temperature dynamics, though formal analysis of diurnal temperature range (DTR) would be required to confirm this pattern. Elevated minimum temperatures during the main growing season could potentially influence respiratory costs and carbon allocation during critical reproductive phases, which in turn may affect bean development and cup quality, though the actual extent of these responses would likely depend on local shade management practices, soil moisture availability, and microclimatic buffering^12^,^10^. The transition from predominantly negative temperature anomalies in the 1990s to consistently positive anomalies in recent decades further suggests that the warming trajectory is embedded within a longer-term climatic shift rather than reflecting short-term oscillatory behavior.

In contrast to the relatively coherent temperature signals, rainfall dynamics in the region appear to be governed more by pronounced interannual variability than by clear long-term directional shifts. Although annual precipitation demonstrates a modest but statistically significant upward trend, this aggregate signal is primarily driven by isolated monthly increases, most notably in September, while the majority of months display non-significant fluctuations. The precipitation regime is further characterized by high coefficients of variation, particularly during the dry and short-rainy months, alongside sharp interannual oscillations between extreme wet and dry years. Such hydroclimatic volatility may present greater challenges for rain-fed agricultural planning than gradual changes in mean totals, as the timing and reliability of moisture availability are critical for flowering induction and fruit maturation in coffee systems. The Precipitation Concentration Index reveals that while annual rainfall distribution remains moderately even, seasonal precipitation during both the Belg and Kiremt periods is highly concentrated. This intensification within shorter temporal windows could enhance surface runoff, modify infiltration dynamics, and increase the likelihood of transient soil moisture deficits during critical phenological stages, even when annual totals remain stable or slightly elevated^17^. The recurring pattern of severe dry periods interspersed with intense wet spells underscores a climate regime that may be growing less predictable, which could complicate traditional farming calendars and alter water balance components essential for crop resilience.

The spatial distribution of climatic trends highlights the influential role of complex topography in moderating local climate responses. The southern portion of the study area consistently records higher seasonal temperatures, whereas northern and eastern zones maintain relatively cooler conditions, a pattern that corresponds with elevation gradients and aspect-driven microclimates. The application of regression kriging, which integrates elevation, slope, and aspect as auxiliary covariates, enabled the capture of these fine-scale variations, reinforcing the observation that climate signals in mountainous coffee-growing regions are rarely uniform. Elevation-mediated temperature gradients may offer some degree of climatic buffering, potentially allowing coffee cultivation to persist at higher altitudes under warming scenarios, although soil quality, land tenure arrangements, and ecological constraints could limit upward expansion^11^. Similarly, rainfall variability demonstrates moderate spatial differentiation across stations, with no single location exhibiting uniformly significant trends, which suggests that local land cover, canopy structure, and orographic effects may interact with broader atmospheric circulation patterns to shape site-specific hydroclimatic conditions. This spatial heterogeneity implies that adaptation strategies relying on uniform assumptions may be less effective, whereas approaches tailored to localized microclimates and landscape positions could offer more meaningful risk reduction.

The co-occurrence of rising temperatures and increasingly erratic rainfall patterns may generate compound stressors that influence both agronomic performance and ecosystem stability. Elevated thermal conditions, when coupled with irregular moisture availability, could alter physiological stress thresholds in coffee plants. Such changes may subsequently affect bean density, biochemical composition, and overall cup quality^40^. Furthermore, shifts in temperature and humidity regimes may influence the geographic ranges and outbreak frequencies of key biotic stressors, including coffee leaf rust (Hemileia vastatrix) and the coffee berry borer (Hypothenemus hampei), whose life cycles are closely tied to microclimatic conditions^13, 41^. While empirical evidence indicates heightened biotic pressure in several tropical regions, the actual impact on yield and farmer livelihoods in the Kafa Zone would likely depend on the adoption of integrated management practices, shade cover density, and local agroecological conditions. The traditional agroforestry systems prevalent in the area, which integrate multiple canopy layers and organic matter inputs, may already provide a degree of microclimatic regulation and hydrological buffering that could mitigate some of these emerging risks^9, 22^. However, the persistence of these systems may be tested if climatic thresholds continue to shift beyond the adaptive capacity of current management frameworks.

In general, the empirical trends documented in this study suggest that the climatic baseline of the Gimbo and Decha districts is transitioning toward a regime characterized by greater thermal stress and hydrological unpredictability^42^. The methodological approach, which combines non-parametric trend detection with innovative trend analysis and spatial interpolation, provides a clear perspective that extends beyond traditional monotonic assessments and captures subtle shifts across different value ranges of climatic variables. These findings align with broader regional assessments while highlighting the importance of ground-validated, district-scale analyses for refining local adaptation planning. Rather than indicating a uniform decline in suitability, the observed patterns point toward a more heterogeneous risk landscape where localized interventions, such as optimized shade management, soil moisture conservation, and diversified livelihood strategies, may help buffer against climatic stressors. Future research would benefit from linking these empirical climate baselines with process-based crop models and participatory vulnerability assessments to better understand how smallholder households perceive and respond to evolving climatic conditions. By grounding adaptation planning in spatially explicit evidence, stakeholders can better design context-sensitive strategies that support both coffee production and the conservation of irreplaceable genetic resources in this biodiverse landscape.

### Conclusions

This study reveals an emerging warming trend across the coffee-producing landscapes of Gimbo and Decha districts in the Kafa Zone from 1990 to 2022. The analysis indicates that a trajectory of moderate but consistent changes, concentrated rainfall patterns, and increasing climate variability. These changes represent a measurable departure from historical climatic norms, with direct implications for the ecological requirements of Coffea arabica cultivation.

The core findings reveal a dual challenge. First, warming during the late dry season (Jan-Mar) may alter bud development and pre-flowering stress thresholds, potentially impacting flowering synchronization when rains arrive. Second, the rainfall regime has become more unstable and concentrated. While annual totals have increased, this gain is characterized by a notable contrast: more intense rainfall events that elevate erosion and waterlogging risks, coupled with a significant decline in dry-season precipitation that may extend periods of moisture deficit. The high Rainfall Concentration Index (PCI) and extreme inter-annual variability underscore a climate of growing unpredictability, which complicates reliable agricultural planning.

Importantly, climatic variability exhibits clear spatial differences across locations. The complex topography creates microclimates where the intensity of trends varies, as demonstrated by the station-level data. This necessitates the development of location-specific adaptation strategies rather than uniform solutions. Nonetheless, the overarching trend across most of the study areas points toward heightened climatic stress.

The implications for the future of coffee farming in this globally significant region are substantial. The combined pressures of rising temperatures and erratic rainfall could threaten not only crop yields and farmer livelihoods but also the conservation of the unique genetic diversity of wild coffee populations in the Kafa Zone. Sustaining this socio-ecological system requires an urgent and integrated response. Adaptation should be grounded in climate-smart agriculture, including resilient shade systems, soil and water conservation techniques, and diversified farming practices. Strengthening institutional support through improved climate information services, tailored financial instruments for smallholders, and robust extension systems is equally crucial.

### Limitations of the study

The author acknowledges some limitations when interpreting the results. First, although the integration of station data with CHIRPS and ERA5-Land improves spatial coverage, these datasets rely on interpolation and may not fully capture fine-scale microclimatic variability in the complex topography of the study area. Second, the temporal coverage (1990-2022) represents a relatively short time series for climate analysis, which may limit the ability to capture long-term variability and multi-decadal climate oscillations, potentially influencing the robustness of detected trends. Third, the study lacks a formal sensitivity analysis to evaluate how methodological choices such as trend detection techniques, interpolation parameters, and seasonal aggregation may influence the results.

Finally, the analysis focuses on climatic trends without directly linking them to crop responses or socio-economic factors, and does not incorporate future climate projections, which may limit its applicability for long-term adaptation planning.

#### Future research directions

Future research should build on the observed warming trend and rainfall variability by integrating these findings into process-based models for *Coffea arabica* to better understand crop responses. Particular attention is needed on soil moisture dynamics and rainfall concentration (PCI > 20), especially under increasing precipitation variability and climate whiplash. The identified spatial heterogeneity across stations highlights the need for fine-scale microclimate monitoring to support location-specific adaptation strategies. In addition, linking the observed climatic changes with pest and disease dynamics, as well as farmer decision-making, would enhance practical relevance. Finally, integrating these historical trends with future climate projections and socio-economic analysis can strengthen long-term adaptation planning.

## Supplementary Information

Supplementary Information.

## Data Availability

The data supporting the findings of this study are available from the corresponding author upon reasonable request.
