# Supplementary material for: Spatiotemporal trends in temperature and rainfall within the coffea arabica landscapes of Gimbo and Decha districts in the Kafa Zone, Southwest Ethiopia
Source: Sci Rep. 2026 May 31;16:24880. doi: 10.1038/s41598-026-54593-y (PMC13457858; doi:10.1038/s41598-026-54593-y)
Supplement: Supplementary file 1 [file 41598_2026_54593_MOESM1_ESM.docx]

**Spatiotemporal Trends in Temperature and Rainfall within the Coffea arabica Landscapes of Gimbo and Decha Districts in the Kafa Zone, Southwest Ethiopia**

Amare Amsalu^1⁎^, Ademe Tizazu^2^, Sintayehu Teka Bedhadha^3^, Dessalegn Obsi Gemeda^4^

1. Department of Natural Resource Management, College of Agriculture and Natural Resource, Bonga University, Bonga, Ethiopia
2. Department of soil science and watershed management, College of Agriculture and Natural Resource, Bonga University, Bonga, Ethiopia
3. Department of Geography and Environmental Studies, College of Social Sciences and Humanities, Jimma University, Jimma, Ethiopia
4. Department of Natural Resource Management, College of Agriculture and Veterinary Medicine, Jimma University, Jimma, Ethiopia

*Corresponding author: [amareamsalunew@gmail.com](mailto:amareamsalunew@gmail.com)

Supplementary Materials (SM)

SM Table1: Innovative Trend Analysis (ITA) Results for Tmax (Monthly and Seasonal)

| Period | ITA Slope (°C/year) | Trend Direction | Δ (°C) Total Change |
| --- | --- | --- | --- |
| Jan | +0.024 | Increasing | +0.79 |
| Feb | +0.014 | Increasing | +0.46 |
| Mar | +0.016 | Increasing | +0.53 |
| Apr | +0.006 | Increasing | +0.20 |
| May | +0.004 | Increasing | +0.13 |
| Jun | +0.003 | Increasing | +0.10 |
| Jul | +0.006 | Increasing | +0.20 |
| Aug | +0.003 | Increasing | +0.10 |
| Sep | +0.004 | Increasing | +0.13 |
| Oct | +0.006 | Increasing | +0.20 |
| Nov | +0.005 | Increasing | +0.17 |
| Dec | +0.006 | Increasing | +0.20 |
| Winter (Bega) | +0.0145 | Increasing | +0.46 |
| Spring (Belg) | +0.009 | Increasing | +0.33 |
| Summer (Kiremt) | +0.004 | Increasing | +0.13 |

SM Table 2: Innovative Trend Analysis (ITA) for Tmin (Monthly and Seasonal)

| Period | ITA Slope (°C/year) | Trend Direction | Δ (°C) Total Change |
| --- | --- | --- | --- |
| Jan | +0.010 | Increasing | +0.33 |
| Feb | +0.012 | Increasing | +0.40 |
| Mar | +0.015 | Increasing | +0.50 |
| Apr | +0.017 | Increasing | +0.56 |
| May | +0.016 | Increasing | +0.53 |
| Jun | +0.021 | Increasing | +0.69 |
| Jul | +0.023 | Increasing | +0.76 |
| Aug | +0.012 | Increasing | +0.40 |
| Sep | +0.010 | Increasing | +0.33 |
| Oct | +0.011 | Increasing | +0.36 |
| Nov | +0.012 | Increasing | +0.40 |
| Dec | +0.010 | Increasing | +0.33 |
| Winter (Bega) | +0.010 | Increasing | +0.36 |
| Spring (Belg) | +0.048 | Increasing | +0.50 |
| Summer (Kiremt) | +0.019 | Increasing | +0.55 |

SM Table 3: Innovative Trend Analysis (ITA) Results for Rainfall (Monthly)

| Month | ITA Slope (mm/year) | Trend Direction | Δ (mm) Total Change |
| --- | --- | --- | --- |
| Jan | -0.18 | Decreasing | -5.94 |
| Feb | +0.12 | Increasing | +3.96 |
| Mar | -0.08 | Decreasing | -2.64 |
| Apr | +0.04 | Increasing | +1.32 |
| May | +0.14 | Increasing | +4.62 |
| Jun | +0.05 | Increasing | +1.65 |
| Jul | +0.06 | Increasing | +1.98 |
| Aug | +0.05 | Increasing | +1.65 |
| Sep | +0.10 | Increasing | +3.30 |
| Oct | +0.03 | Increasing | +0.99 |
| Nov | +0.11 | Increasing | +3.63 |
| Dec | -0.09 | Decreasing | -2.97 |

### SM **Table 4: Innovative Trend Analysis (ITA) results for rainfall (1990-2022)**

| Category | ITA Slope (mm/year) | Trend Direction | Δ (mm) Total Change |
| --- | --- | --- | --- |
| Low (10^th^-33rd percentile) | +0.084 | Increasing | +2.77 |
| Medium (34^th^-66th percentile) | +0.033 | Increasing | +1.09 |
| High (67^th^-90th percentile) | +0.072 | Increasing | +2.38 |
| Annual (all values) | +0.064 | Increasing | +2.11 |

SM Table 5: Station coordinates, elevation, and data periods

| No. | Station | Latitude | Longitude | Altitude (m) | Data Period |
| --- | --- | --- | --- | --- | --- |
| 1 | Chira | 7.44 | 36.14 | 2095 | 1990-2022 |
| 2 | Bitawoshi | 7.19 | 36.02 | 1836 | 1990-2022 |
| 3 | Kemise | 7.19 | 36.08 | 1950 | 1990-2022 |
| 4 | Wush Wush | 7.18 | 36.08 | 1620 | 1990-2022 |
| 5 | Keresi | 7.13 | 36.14 | 1650 | 1990-2022 |
| 6 | Sheda Tura | 7.16 | 36.23 | 1745 | 1990-2022 |
| 7 | Goga Kemise | 7.25 | 36.23 | 1250 | 1990-2022 |
| 8 | Bonga | 7.30 | 36.30 | 1779 | 1990-2022 |
| 9 | Seka Chekorsa | 7.33 | 36.33 | 1919 | 1990-2022 |
| 10 | Yebu | 7.68 | 36.86 | 1982 | 1990-2022 |

SM Table 6: Mean maximum temperature at seasonal scale (1990-202022).

| Station | Summer (Zs, p, Sen’s) | Autumn (Zs, p, Sen’s) | Winter (Zs, p, Sen’s) | Spring (Zs, p, Sen’s) |
| --- | --- | --- | --- | --- |
| Chira | 1.70, 0.06, 0.01 | *2.37, 0.02, 0.02 | *2.89, 0.004, 0.03 | *2.74, 0.01, 0.03 |
| Bitawoshi | *3.90, 0.001, 0.04 | *3.21, 0.002, 0.04 | *3.85, 0.001, 0.05 | *4.10, 0.001, 0.05 |
| Kemise | *2.05, 0.04, 0.01 | 0.78, 0.27, 0.01 | 1.12, 0.14, 0.01 | 1.40, 0.16, 0.01 |
| Wush Wush | *3.45, 0.001, 0.03 | *2.30, 0.02, 0.02 | *3.60, 0.001, 0.04 | *3.05, 0.002, 0.04 |
| Keresi | 1.86, 0.06, 0.01 | 0.75, 0.45, 0.00 | 0.74, 0.46, 0.01 | 0.63, 0.52, 0.01 |
| Sheda Tura | *3.98, 0.001, 0.04 | 0.47, 0.64, 0.00 | 1.58, 0.11, 0.01 | 1.71, 0.09, 0.02 |
| Goga Kemise | *3.60, 0.001, 0.04 | *3.42, 0.001, 0.04 | *4.15, 0.001, 0.05 | *3.88, 0.001, 0.05 |
| Bonga | *4.25, 0.001, 0.05 | *3.06, 0.002, 0.04 | *4.80, 0.001, 0.05 | *4.45, 0.001, 0.05 |
| Seka Chekorsa | *4.10, 0.001, 0.05 | *3.78, 0.001, 0.04 | *4.50, 0.001, 0.05 | *4.20, 0.001, 0.05 |
| Yebu | *4.85, 0.001, 0.05 | *4.20, 0.001, 0.05 | *4.60, 0.001, 0.05 | *4.35, 0.001, 0.05 |

Note: *Zs > 1.96 (α = 0.05) indicates statistical significance at the 5% level.

SM Table 7: Mean Minimum Temperature at Seasonal Scale (1990-202022).

| Stations | Summer (Zs, p, Sen’s) | Autumn (Zs, p, Sen’s) | Winter (Zs, p, Sen’s) | Spring (Zs, p, Sen’s) |
| --- | --- | --- | --- | --- |
| Chira | 0.51, 0.61, 0.01 | 1.74, 0.08, 0.01 | 0.78, 0.43, 0.01 | 0.71, 0.48, 0.01 |
| Bitawoshi | *3.10, 0.002, 0.03 | 0.28, 0.78, 0.00 | 0.46, 0.65, 0.00 | 0.04, 0.96, 0.00 |
| Kemise | *3.45, 0.001, 0.03 | *3.05, 0.002, 0.03 | *3.20, 0.001, 0.03 | *2.95, 0.003, 0.03 |
| Wush Wush | *3.16, 0.002, 0.03 | *3.21, 0.001, 0.02 | 1.39, 0.16, 0.01 | *3.85, 0.001, 0.03 |
| Keresi | 1.38, 0.17, 0.02 | 1.74, 0.08, 0.01 | 1.58, 0.11, 0.02 | 1.27, 0.20, 0.01 |
| Sheda Tura | *3.05, 0.002, 0.02 | *4.10, 0.001, 0.02 | *3.45, 0.001, 0.03 | 0.27, 0.79, 0.00 |
| Goga Kemise | *3.60, 0.001, 0.03 | *3.42, 0.001, 0.03 | *3.09, 0.002, 0.03 | *3.50, 0.001, 0.03 |
| Bonga | *4.85, 0.001, 0.04 | *4.60, 0.001, 0.04 | *4.15, 0.001, 0.04 | *3.95, 0.001, 0.04 |
| Seka Chekorsa | *4.70, 0.001, 0.04 | *4.95, 0.001, 0.04 | *3.61, 0.001, 0.03 | *4.20, 0.001, 0.04 |
| Yebu | *2.40, 0.02, 0.02 | 1.15, 0.25, 0.01 | 0.40, 0.69, 0.01 | 1.78, 0.08, 0.01 |

Note: *Zs > 1.96 (α = 0.05) indicates statistical significance at the 5% level.

SM Table 8: Validation Metrics for CHIRPS Precipitation vs. Station Observations (1990-2022)

| Temporal Scale | *r* | RMSE (mm) | MAE (mm) | PBIAS (%) | NSE | Sample Size (*n*) |
| --- | --- | --- | --- | --- | --- | --- |
| Annual | 0.82 | 156 | 112 | +3.2 | 0.71 | 330 |
| Kiremt (Jun-Aug) | 0.86 | 48 | 35 | +4.1 | 0.79 | 1,320 |
| Belg (March-May) | 0.71 | 38 | 29 | -2.8 | 0.64 | 1,320 |
| Dry Season (Dec-Feb) | 0.68 | 22 | 17 | -5.3 | 0.58 | 1,320 |
| Monthly (all months) | 0.74 | 31 | 24 | +1.1 | 0.66 | 3,960 |

*Note: r = Pearson correlation; RMSE = Root Mean Square Error; MAE = Mean Absolute Error; PBIAS = Percent Bias; NSE = Nash-Sutcliffe Efficiency. Positive PBIAS indicates overestimation by CHIRPS.*

SM Table 9: Validation Metrics for ERA5-Land Temperature vs. Station Observations (1990-2022)

| Variable | Temporal Scale | *r* | RMSE (°C) | MAE (°C) | PBIAS (°C) | NSE | Sample Size (*n*) |
| --- | --- | --- | --- | --- | --- | --- | --- |
| Tmean | Annual | 0.94 | 0.87 | 0.68 | -0.3 | 0.89 | 330 |
|  | Seasonal | 0.93 | 0.91 | 0.71 | -0.2 | 0.87 | 1,320 |
|  | Monthly | 0.92 | 0.95 | 0.74 | -0.1 | 0.85 | 3,960 |
| Tmax | Annual | 0.91 | 1.12 | 0.89 | -0.5 | 0.85 | 330 |
|  | Seasonal | 0.89 | 1.18 | 0.94 | -0.6 | 0.82 | 1,320 |
|  | Monthly | 0.88 | 1.24 | 0.98 | -0.4 | 0.80 | 3,960 |
| Tmin | Annual | 0.96 | 0.71 | 0.54 | +0.2 | 0.92 | 330 |
|  | Seasonal | 0.95 | 0.76 | 0.58 | +0.3 | 0.91 | 1,320 |
|  | Monthly | 0.94 | 0.81 | 0.62 | +0.1 | 0.89 | 3,960 |

SM Table 10: Station-Level Validation Performance for CHIRPS Precipitation (Annual Scale)

| Station | *r* | RMSE (mm yr⁻¹) | PBIAS (%) | NSE | Performance Rating |
| --- | --- | --- | --- | --- | --- |
| Chira | 0.79 | 168 | +4.8 | 0.68 | Good |
| Bitawoshi | 0.74 | 182 | +6.2 | 0.62 | Good |
| Kemise | 0.81 | 151 | +2.9 | 0.73 | Good |
| Wush Wush | 0.72 | 189 | +7.1 | 0.59 | Moderate |
| Keresi | 0.85 | 142 | +1.8 | 0.76 | Very Good |
| Sheda Tura | 0.83 | 148 | +3.1 | 0.74 | Good |
| Goga Kemise | 0.84 | 145 | +2.5 | 0.75 | Good |
| Bonga | 0.77 | 172 | +5.4 | 0.65 | Good |
| Seka Chekorsa | 0.80 | 159 | +3.7 | 0.70 | Good |
| Yebu | 0.86 | 138 | +1.2 | 0.78 | Very Good |
| Regional Mean | 0.80 | 159 | +3.9 | 0.70 | Good |

*Note: Performance rating based on NSE: ≥ 0.75 = Very Good; 0.65-0.74 = Good; 0.50-0.64 = Moderate; <0.50 = Poor (Moriasi et al., 2007).*

SM Table 11: Leave-One-Out Cross-Validation (LOOCV) for Regression Kriging Interpolation

| Variable | Metric | LOOCV Result | Interpretation |
| --- | --- | --- | --- |
| Precipitation (CHIRPS) | RMSE | 142 mm yr⁻¹ | Acceptable for regional trend analysis |
|  | MAE | 98 mm yr⁻¹ | Consistent with station density |
|  | R² | 0.78 | Strong spatial predictive capacity |
|  | Median Absolute Error | 89 mm yr⁻¹ | Robust to extreme values |
| Temperature (ERA5-land) | RMSE (Tmean) | 0.90 °C | High accuracy for trend detection |
|  | MAE (Tmean) | 0.70 °C | Minimal systematic bias |
|  | R² (Tmean) | 0.94 | Excellent spatial representation |
|  | RMSE (Tmax/Tmin) | 1.18 / 0.76 °C | Slightly higher uncertainty for daytime extremes |

SM Table 12: Symbol Legend

| Symbol | Definition | Units/Notes |
| --- | --- | --- |
| *n* | Number of observations in time series | dimensionless |
| *Xᵢ*, *Xⱼ*, *Xₖ* | Climate variable value at time step *i*, *j*, *k* | °C or mm |
| *S* | Mann-Kendall test statistic | dimensionless |
| *Var(S)* | Variance of *S*, adjusted for tied groups | dimensionless |
| *Zₛ* | Standardized MK test statistic | dimensionless; |
| *Qᵢ* | Individual slope estimate for pair (*j*, *k*) | °C yr⁻¹ or mm yr⁻¹ |
| *Q̃* | Sen's slope estimator (median of all *Qᵢ*) | °C yr⁻¹ or mm yr⁻¹ |
| *σ* | Standard deviation of rainfall time series | mm |
| *μ* | Long-term mean of rainfall time series | mm |
| CV | Coefficient of Variation | % |
| SRAᵢ | Standardized Rainfall Anomaly for year *i* | dimensionless (Z-score) |
| *t* | Number of tied groups in data | dimensionless |
| tᵢ | Size of the *i*th tied group | dimensionless |

SM Figure 1: Elevation map of the study area

SM Figure 2: Station locations in the study area

SM Figure 3: Spatial distributions of minimum annual temperature

SM Figure 4: Spatial distributions of maximum annual temperature

SM Figure 5: Spatial distributions of mean annual temperature

SM Figure 6: Spatial distribution monthly rainfall
